# A wheat kinase and immune receptor form host-specificity barriers against the blast fungus

**DOI:** 10.1038/s41477-023-01357-5

**Published:** 2023-02-16

**Authors:** Sanu Arora, Andrew Steed, Rachel Goddard, Kumar Gaurav, Tom O’Hara, Adam Schoen, Nidhi Rawat, Ahmed F. Elkot, Andrey V. Korolev, Catherine Chinoy, Martha H. Nicholson, Soichiro Asuke, Rea Antoniou-Kourounioti, Burkhard Steuernagel, Guotai Yu, Rajani Awal, Macarena Forner-Martínez, Luzie Wingen, Erin Baggs, Jonathan Clarke, Diane G. O. Saunders, Ksenia V. Krasileva, Yukio Tosa, Jonathan D. G. Jones, Vijay K. Tiwari, Brande B. H. Wulff, Paul Nicholson

**Affiliations:** 1 John Innes Centre, Norwich Research Park, Norwich, UK; 2 Department of Plant Science and Landscape Architecture, University of Maryland, College Park, MD USA; 3 Wheat Research Department, Field Crops Research Institute, Agricultural Research Center, Giza, Egypt; 4 Graduate School of Agricultural Science, Kobe University, Kobe, Japan; 5 Department of Plant and Microbial Biology, University of California, Berkeley, CA USA; 6 The Sainsbury Laboratory, Norwich Research Park, Norwich, UK; 7 Present Address: Limagrain UK Ltd, Lincolnshire, UK; 8 Present Address: European Molecular Biology Laboratory, European Bioinformatics Institute EMBL-EBI, Hinxton, UK; 9 Present Address: School of Molecular Biosciences, College of Medical, Veterinary and Life Sciences, University of Glasgow, Glasgow, UK; 10 Present Address: Center for Desert Agriculture, Biological and Environmental Science and Engineering Division (BESE), King Abdullah University of Science and Technology (KAUST), Thuwal, Saudi Arabia

**Keywords:** Plant genetics, Plant immunity, Plant breeding

## Abstract

Since emerging in Brazil in 1985, wheat blast has spread throughout South America and recently appeared in Bangladesh and Zambia. Here we show that two wheat resistance genes, *Rwt3* and *Rwt4*, acting as host-specificity barriers against non-*Triticum* blast pathotypes encode a nucleotide-binding leucine-rich repeat immune receptor and a tandem kinase, respectively. Molecular isolation of these genes will enable study of the molecular interaction between pathogen effector and host resistance genes.

## Abstract (web summary)

This study identifies two genes that are important for protecting wheat against blast fungus, a pathogen that also infects related grasses. This work helps identify potential strategies for breeding wheat with improved resistance to this damaging fungus.

## Main

Wheat blast, caused by *Pyricularia oryzae* (syn. *Magnaporthe oryzae*) pathotype *Triticum* was first identified in Brazil in 1985 (ref. ^1^). The pathogen subsequently spread to cause epidemics in other regions of Brazil and neighbouring countries, including Bolivia and Paraguay^2^. Outbreaks of wheat blast occurred in Bangladesh in 2016, and the disease was reported from Zambia in 2018 (refs. ^3,4^). Wheat blast is now considered to pose a threat to global wheat production^5^, and discovery and deployment of resistance genes against this pathogen are critical to mitigate its threat.

While *P. oryzae* exhibits a high level of host specificity, *Triticum* pathotypes are closely related to *Lolium* pathotypes^6^. Two pathogen genes, *PWT3* and *PWT4*, condition avirulence of different isolates of *P. oryzae* pathotype *Lolium* on wheat (*Triticum aestivum*).

The resistance genes *Rwt3* and *Rwt4* in wheat recognize respectively the *PWT3* and *PWT4* avirulence gene products to prevent infection. It was originally proposed that the epidemics in Brazil occurred due to the widespread cultivation of varieties lacking *Rwt3* that are susceptible to *Lolium* pathotypes^6^. *Lolium* pathotypes have also been associated with the occurrence of wheat blast in the United States^7,8^. Recent studies suggest that both *Triticum* and *Lolium* pathotypes emerged as part of a multi-hybrid swarm in which key host specificity determinants were re-assorted^9^.

**Fig. 1:**
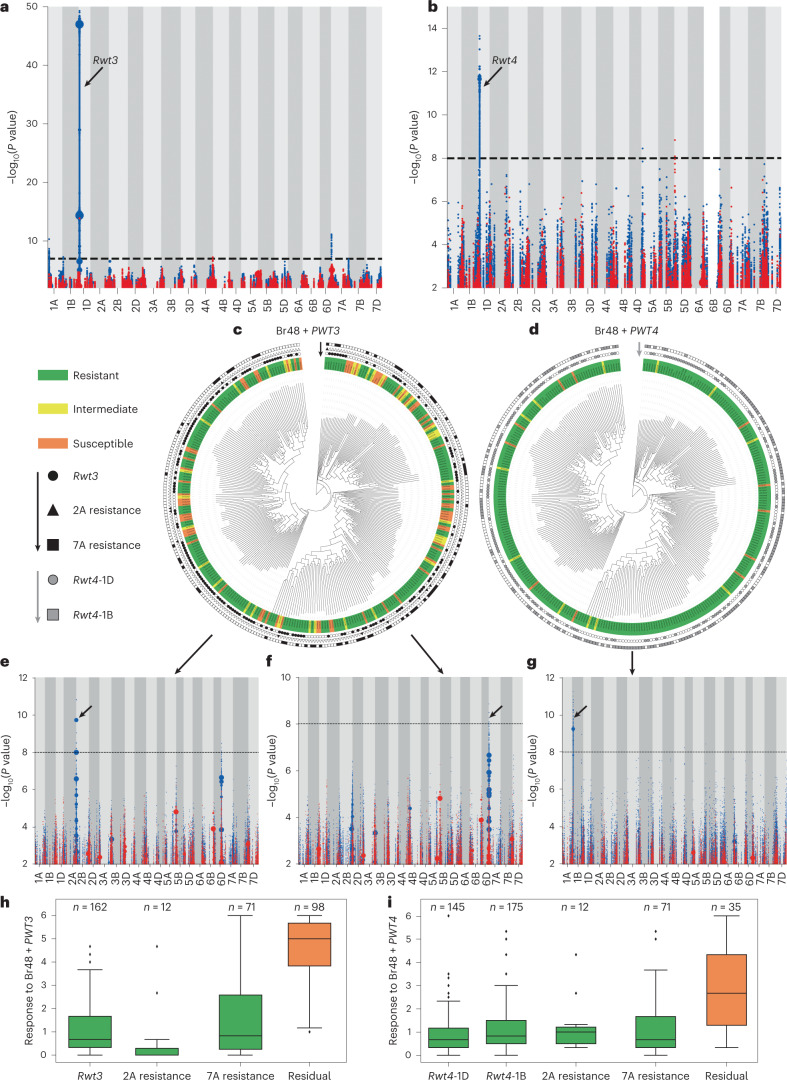
Genetic identification of resistances to the blast fungus by *k*-mer-based association mapping on an *R*-gene enriched sequencing panel of wheat landraces. **a**,**b**, *k*-mers associated with resistance to (Br48 + *PWT3* mapped to Chinese Spring (**a**) and Br48 + *PWT4* mapped to Jagger (**b**). **c**,**d**, *k*-mer-based phylogeny of wheat landraces showing the phenotype of an accession after inoculation with: Br48 + *PWT3* (**c**) and Br48 + *PWT4* (**d**), and the predicted presence of the putative resistances. Phenotype of an accession after inoculation with a blast isolate is indicated by the colour used to highlight the label of that accession, while the presence and absence of allele-specific polymorphisms is indicated by filled symbols with black/grey and white, respectively. **e**,**f**, *k*-mers significantly associated with resistance to Br48 + *PWT3* in the absence of the *Rwt3* candidate gene on chromosome 1D leads to the identification of a resistance on chromosome 2A when mapped to the assembly of wheat cultivar SY Mattis (**e**), and chromosome 7A when mapped to wheat cultivar Jagger (**f**). **g**, *k*-mers significantly associated with resistance to Br48 + *PWT4* in the absence of *Rwt4* candidate gene on chromosome 1D leads to the identification of a resistance on a region of chromosome 1B containing the homoeologue of *Rwt4* when mapped to Jagger. **h**, Box plots showing variation for resistance to Br48 + *PWT3* in Watkins lines predicted to carry *Rwt3*, 2A resistance, 7A resistance and none of them. **i**, Box plots showing variation for resistance to Br48 + *PWT4* in Watkins lines predicted to carry *Rwt4*-1D*, Rwt4*-1B, 2A resistance, 7A resistance and none of them. In the box plots of **h** and **i**, boxes denote the interquartile range with the horizontal bar inside representing the median. Whiskers extend to 1.5 times the interquartile range, values outside of which are considered outliers and shown as individual points. Number of Watkins lines belonging to each class is indicated at the top of the corresponding box plot. In the association plots of **a** and **b** and **e**–**g**: points on the *y* axis depict *k*-mers positively associated with resistance in blue and negatively associated with resistance in red. Point size is proportional to the number of *k*-mers. The association score is defined as the –log_10_ of the *P* value obtained using the likelihood ratio test for nested models. The threshold of significant association scores is adjusted for multiple comparisons using the Bonferroni approximation. Arrows indicate regions with significant association scores.

In this Brief Communication, to identify candidates for *Rwt3* and *Rwt4*, we used a Triticeae bait library (Supplementary Table 1 and Additional File 1) to capture and sequence the nucleotide-binding site leucine-rich repeat protein (NLR) complements of 320 wheat lines including 300 wheat landraces from the A.E. Watkins collection harbouring the genetic diversity existing before intensive breeding (Supplementary Table 2 and Supplementary Fig. 1). We screened seedlings of the panel with Br48, a *Triticum* pathotype strain of *P. oryzae*, transformed with either *PWT3* or *PWT4* (ref. ^6^) (Supplementary Table 3 and Supplementary Figs. 2 and 3) and performed *k*-mer-based association genetics using Chinese Spring^10^ and Jagger^11^ as the reference genomes, respectively. This led to identification of candidate NLR genes, *TraesCS1D02G029900* and *TraesJAG1D03G00423690*, for *PWT3* and *PWT4* recognition (Fig. 1a,b and Supplementary Figs. 4 and 5) on chromosome 1D within the previously defined biparental mapping intervals of *Rwt3* and *Rwt4*, respectively^12,13^.

**Fig. 2:**
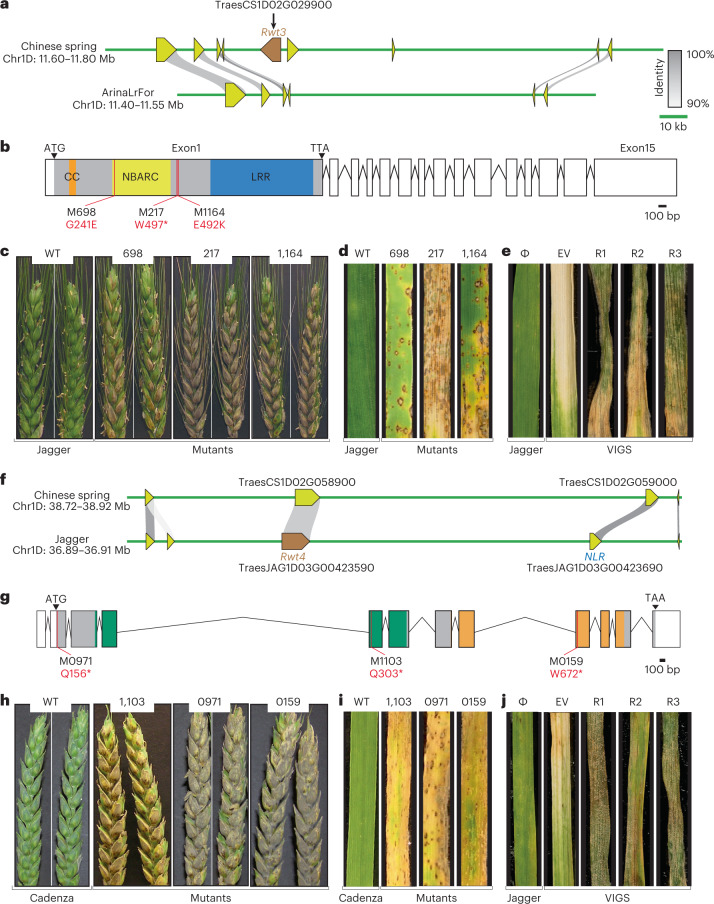
Genetic and functional characterization of *Rwt3* and *Rwt4*. **a**, Gene-based collinearity analysis of the two haplotypes linked to *Rwt3* identified in the wheat pangenome. **b**, Structure of the NLR candidate gene for *Rwt3*. The predicted 1,069-amino-acid protein has domains with homology to a CC, NB-ARC and LRRs. **c**,**d**, Wheat blast head (**c**) and detached leaf (**d**) assays for the *Rwt3* Jagger mutants and wild type with Br48 + *PWT3*. **e**, Leaf segments from plants subjected to VIGS with non-virus control (Φ), empty vector (EV) and *Rwt3* target (R1, R2 and R3) and super-infected with Br48 + *PWT3*. **f**, Gene-based collinearity analysis of the two haplotypes linked to *Rwt4* identified in the wheat pangenome. **g**, Structure of the WTK candidate gene for *Rwt4*. The predicted protein of 895 amino acids has domains with homology to a WTK (shown with green and orange colours). **h**,**i**, Wheat blast head (**h**) and detached leaf (**i**) assays for the *Rwt4* Cadenza mutants and wild type with Br48 + *PWT4*. **j**, Leaf segments from plants subjected to VIGS with non-virus control (Φ), empty vector (EV) and *Rwt4* target (R1, R2 and R3) and super-infected with Br48 + *PWT4*.

To investigate the allelic variation of these candidate genes, we used BLASTn (ref. ^14^) to query their sequences in the NLR assemblies of *Aegilops tauschii*^15^, the D-genome progenitor of bread wheat and Watkins wheat landraces. For *Rwt3* NLR candidate, we identified four groups of allelic variants as well as presence/absence variation in *Ae. tauschii*; however, only presence/absence variation was observed in wheat landraces (Fig. 2a, Additional File 2, Supplementary Table 4 and Supplementary Fig. 6). For *Rwt4* NLR candidate, we identified five groups of allelic variants in *Ae. tauschii* out of which only two were observed in wheat landraces (Fig. 2f, Additional File 3, Supplementary Table 4 and Supplementary Fig. 6). Although *Rwt4* NLR candidate is allelic to an NLR in linkage with *Pm24* (ref. ^16^), we did not find *Pm24* linked NLR in either *Ae. tauschii* or Watkins landraces, which is consistent with the post-domestication origin hypothesis of *Pm24* in China^16^. *Rwt4* candidate was found only in lineage 2 (L2) of *Ae. tauschii*, while *Rwt3* candidate was found only in lineage 1 (L1), which explains why we could identify only the *Rwt4* candidate, and not the *Rwt3* candidate, by phenotyping and performing association genetics on an NLR gene enrichment-sequenced *Ae. tauschii* L2 panel^15^ (Supplementary Table 5 and Supplementary Figs. 6–8). The L1 origin of *Rwt3* is remarkable considering that the L1 signature in wheat is mostly concentrated around a 5 Mb region surrounding the *Rwt3* candidate^17^ (Supplementary Fig. 9). This finding suggests that interaction with pathogens may have played a role in wheat evolution.

To functionally validate the *Rwt3* NLR candidate, we screened a TILLING population of Jagger^18^ and found three lines each carrying a functional mutation in this gene (Supplementary Fig. 10). One line, M217, is homozygous for a mutation causing a premature stop codon, whereas another, M698, is homozygous for a mis-sense mutation (G241E) predicted to cause functional aberration in the protein (Fig. 2b and Supplementary Table 6). In both the leaf and head assays of these mutants using Br48 + *PWT3*, a loss of the wild-type resistance was observed (Fig. 2c,d). The third line, M1164, is heterozygous for another deleterious mis-sense mutation (E492K) (Fig. 2b and Supplementary Table 6). In both the leaf and head assays of the segregating progeny of M1164 using Br48 + *PWT3*, those homozygous for the mutation were found to be susceptible while the others were resistant (Fig. 2c,d and Supplementary Fig. 11). Virus-induced gene silencing (VIGS) was performed to confirm the role of the *Rwt3* NLR candidate. Resistance to Br48 + *PWT3* was lost in wheat cv. Jagger following silencing with barley stripe mosaic virus (BSMV) expressing a 400 bp *Rwt3* gene fragment (Fig. 2e and Supplementary Fig. 12). The clear loss of function observed in three independently derived TILLING mutants, the co-segregation of the M1164 mutation with susceptibility and the effect of VIGS show that the *Rwt3* NLR candidate is required for resistance to *P. oryzae* expressing the *PWT3* effector.

As remarked earlier, the identified *Rwt4* NLR candidate is adjacent to an allele of a wheat tandem kinase (WTK), designated *Pm24*, which confers resistance against powdery mildew^16^ (Fig. 2f). Therefore, we tested both the identified *Rwt4* NLR candidate (Supplementary Fig. 5) and the linked *Pm24* allele, *TraesJAG1D03G00423590* (ref. ^11^) (Supplementary Fig. 13), as candidates for *Rwt4* using the Cadenza TILLING resource^19^. For the NLR candidate, we tested four lines (two heterozygous and two homozygous) carrying mutations predicted to cause premature stop codons and three additional lines (two heterozygous and one homozygous) carrying mis-sense mutations predicted to have a significant impact on tertiary structure (Supplementary Fig. 5a and Supplementary Table 6). Neither the homozygous nor any progeny of the heterozygous mutants for this candidate showed an increase in susceptibility relative to the wild type Cadenza in either leaf or head assays with Br48 + *PWT4* (Supplementary Fig. 14). For the linked *Pm24* allele, we tested three lines (one homozygous and two heterozygous) carrying mutations that result in premature stop codons (Fig. 2g and Supplementary Table 6). In both the leaf and head assays of the homozygous line M0159 using Br48 + *PWT4*, a clear increase in susceptibility compared with the wild type was observed (Fig. 2h,i). In the leaf and head assays of the segregating progeny of heterozygous mutants (M0971 and M1103) using Br48 + *PWT4*, those homozygous for the mutation were found to be susceptible while all others were resistant (Fig. 2h,i and Supplementary Fig. 15). VIGS was performed to confirm the role of the *Rwt4* WTK candidate. Resistance to Br48 + *PWT4* was lost in wheat cv. Jagger following silencing with a 400 bp gene fragment of *Rwt4* WTK (Fig. 2j and Supplementary Fig. 16). These results show that the linked WTK, and not the identified NLR candidate, is required for resistance to *P. oryzae* expressing the *PWT4* effector. The finding that *WTK* alleles, *Pm24* and *Rwt4*, are involved in resistance to two unrelated fungal pathogens suggests that it may be a broad-spectrum component of disease resistance.

We developed Kompetitive Allele-Specific PCR (KASP) markers for *Rwt3* and *Rwt4* (Supplementary Table 7) and tested them on the core 300 Watkins lines obtaining a validation rate of 97% and 99% based on their corresponding in silico predictions (Supplementary Table 8). Based on the in silico predictions, *Rwt3* is present only in 148 of the 192 Watkins lines resistant to Br48 + *PWT3* (Fig. 1c and Supplementary Table 8), while *Rwt4* is present in only 141 of the 271 Watkins lines resistant to Br48 + *PWT4* (Fig. 1d and Supplementary Table 8). This suggests that there are other resistance genes in the Watkins panel recognizing *PWT3*, *PWT4* or additional effectors in Br48. We re-ran GWAS with the leaf assay disease phenotype of Br48 + *PWT4*, restricted to the Watkins lines not containing *Rwt4*. Using Jagger^11^ as the reference genome, we obtained a clear peak on chromosome 1B in the region homoeologous to that on 1D containing *Rwt4* (Fig. 1g), indicating that *Rwt4* has a homoeologue on chromosome 1B that provides resistance to *P. oryzae* expressing the *PWT4* effector. We followed the same protocol and re-ran the GWAS with the leaf assay disease phenotype of Br48 + *PWT3*, restricted to the Watkins lines not containing *Rwt3*. This identified a clear peak on chromosome 2A using Mattis^11^ as the reference genome (Fig. 1e) and another on chromosome 7A using Jagger^11^ as the reference genome (Fig. 1f). A resistance termed *Rmg2* located on chromosome 7A has previously been identified in the cultivar Thatcher^20^ and a resistance termed *Rmg7* has been reported on the distal region of the long arm of chromosome 2A of tetraploid wheat^21^. In both instances the resistances were identified using the same isolate, Br48, as used in our work suggesting that the resistances identified on chromosomes 2A and 7A may correspond to *Rmg7* and *Rmg2* reported previously. Watkins lines carrying either of these two resistance loci showed resistance to both Br48 + *PWT3* and Br48 + *PWT4* (Fig. 1h,i). Additionally, Watkins lines carrying the 7A resistance showed similar levels of resistance to both Br48 and Br48 + *PWT3* (Supplementary Fig. 17). These observations suggested that the 7A and 2A resistance loci interact with Br48 and supported their characterization as *Rmg2* and *Rmg7*, respectively. Based on the in silico predictions, at least one of *Rwt3*, 2A and 7A resistance loci is present in 177 of the 192 Watkins lines resistant to Br48 + *PWT3* (Fig. 1c and Supplementary Table 8), while at least one of *Rwt4-*1D, *Rwt4-*1B, 2A and 7A resistance loci is present in 251 of the 271 Watkins lines resistant to Br48 + *PWT4* (Fig. 1d and Supplementary Table 8).

We designed a KASP marker for the *Rwt4*-1B homoeologue (Supplementary Tables 7 and 9) that, along with those for *Rwt3* and *Rwt4*-1D, should enable wheat breeders to ensure that cultivars maintain host-specificity barriers. It was recently shown that, even though the pandemic clonal lineages tend to dominate *Triticum* pathotypes, sexual recombination between *Triticum* and non-*Triticum* pathotypes is possible and can raise the adaptive potential of *Triticum* pathotypes in the absence of host-specificity barriers^22,23^. This suggests that *PWT4*, which is naturally found in *Lolium* pathotypes isolated from *Avena* but not in *Triticum* pathotypes^6^, can potentially be gained by *Triticum* pathotypes through sexual recombination. Gain of *PWT4* by *Triticum* pathotypes would suppress the resistance conferred by *Rmg8* against *AVR-Rmg8* in the absence of *Rwt4* (ref. ^24^) (Supplementary Fig. 18). Therefore, the possibility of either gain of *PWT4* or the loss-of-function mutations in *AVR-Rmg8* threatens one of the few reported resistances that show effectiveness against *Triticum* pathotypes at both the seedling and head stage^25^.

So far, 11 postulated resistance genes against *P. oryzae* have been identified in wheat and none of these has been cloned^26^. We used isolates of *P. oryzae* differing in a single effector to screen genome sequenced/characterized diversity panels of *Ae. tauschii* and hexaploid wheat to clone *Rwt3* and *Rwt4* resistance genes that confer resistance in wheat to *P. oryzae* isolates carrying *PWT3* and *PWT4*, respectively. The isolation of effector and resistance gene pairs will enable study of their interaction and potential for engineering as well as provide leads to identify genes effective against the *Triticum* pathotype of *P. oryzae*.

## Methods

### Watkins panel configuration

Using the SSR genotype data from Wingen et al. (2014) (ref. ^27^), a core set of 300 genetically diverse wheat landraces with spring growth habit were selected from the Watkins collection (Supplementary Fig. 1 and Supplementary Table 2) along with 20 non-Watkins lines. The DNA was extracted following a modified CTAB protocol^28^. The seeds of these lines are available from the Germplasm Resources Unit (www.seedstor.ac.uk) under wheat resistance gene enrichment (WREN) sequencing collection (WREN0001- WREN0320).

### Phenotyping of *Ae. tauschii* and Watkins panels with wheat blast isolates

The *M. oryzae* pathotype *Triticum* (MoT) isolate Br48 and the transformed isolates Br48 + *PWT3* and Br48 + *PWT4* (ref. ^6^) were grown on complete medium agar. A conidial suspension of 0.3–0.4 × 10^6^ conidia per millilitre was used for all inoculations. Detached seedling assays with the *Ae. tauschii* and Watkins panels were carried out as described by Goddard et al. (2020) (ref. ^29^) and scored for disease symptoms using a 0–6 scale (Supplementary Figs. 2, 3 and 7 and Supplementary Tables 3 and 5). Resistance at the heading stage was assessed according to Goddard et al. (2020) (ref. ^29^). Heads of *Ae. tauschii* and wheat were scored using a 0–6 scale (Supplementary Fig. 2e and 2f, respectively).

### Bait library design for the Watkins panel

Two bait libraries were used for the capture of the immune receptors from the Watkins panel (1) NLR Triticeae bait library V3 (https://github.com/steuernb/MutantHunter/), including 275 genes conserved in grasses^30^ and (2) a new bait library that included NLRs extracted from the genomes of *T. turgidum* cv. Svevo and cv. Kronos and *T. dicoccoides* cv. Zavitan, and only those genes that had <50% coverage by previously designed baits were used. To remove redundancies, NLR sequences were passed through CD-HIT (v4.6.8-2017-0621 -c 0.9 -G 0 -aS 0.9 -p 1). This bait design also included wheat domestication genes *VRN1A* (AY747598), *Wx1* (AY050174), *Q* (AY702956), *Rht-b1* (JX993615), *Rht-d1* (HE585643), *NAM-B1* (MG587710) and wheat orthologues of known immune signalling components ICS1, NPR1, NDR1, EDS1, PAD4, SRFR1, SAG101, RAR1, SGT1, HSP90.2, HSP90.4, RIN4, ADR1 and PBS1 extracted through BioMart (Supplementary Table 1 and Additional File 1). The bait probes were designed by Arbor Bioscience and filtered with their Repeat Mask pipeline, which removed the baits that were >50% repeat masked and any non-NLR baits with more than three hits in the wheat genome. To balance for the low copy number genes, baits derived from domestication genes were multiplied 10× and those derived from immune signalling genes were 3× compared with the baits derived from NLRs.

### Library construction and sequencing of the Watkins panel

Illumina libraries with an average insert size of 700 bp were enriched by Arbor Biosciences, as previously described^31^, and sequenced on an Illumina HiSeq with either 150 or 250 paired end (PE) reads at Novogene, China to generate an average of 3.82 Gb per accession (Supplementary Table 2). The raw reads were trimmed using Trimmomatic v0.2 (ref. ^32^) and de novo assembled with the CLC Assembly Cell (http://www.clcbio.com/products/clc-assembly-cell/) using word size (-w = 64) with standard parameters.

### Generating Watkins *k*-mer presence/absence matrix and its phylogeny

A presence/absence matrix of *k*-mers (*k* = 51) was constructed from trimmed raw data using Jellyfish^33^ as described in Arora et al. (2019) (ref. ^15^). *k*-mers occurring in fewer than four lines or in all but three or fewer lines were removed during the construction of the matrix. From the *k*-mer matrix generated with Watkins RenSeq data, 5,310 randomly extracted *k*-mers were used to build an unweighted pair group method with arithmetic mean (UPGMA) tree with 100 bootstraps.

### *k*-mer-based association mapping

For the reference genomes of *T. aestivum*—Chinese Spring^10^, Jagger^11^ and Mattis^11^—and of *Ae. tauschii* AY61 (ref. ^34^), NLRs were predicted using NLR-Annotator^35^ and their sequences along with 3 kb sequence from both upstream and downstream region (if available) were extracted using samtools (version 1.9) to create the corresponding reference NLR assemblies. The disease phenotypes were averaged across the replicates after removing the non-numerical values and the mean phenotype scores multiplied by −1 so that a higher value represents a higher resistance. For those *k*-mers of a reference NLR assembly whose presence/absence in the panel correlates with the phenotype, that is, the absolute value of Pearson’s correlation obtained was higher than 0.1, a *P* value was assigned using linear regression while taking the three most significant principal component analysis dimensions as covariates to control for the population structure. A stringent cut-off of 8, based on Bonferroni adjustment^17^ to a *P* value of 0.05, was chosen for Watkins RenSeq association mapping (Fig. 1), while a cut-off of 7 was chosen for *Ae. tauschii* L2 RenSeq association mapping (Supplementary Fig. 8).

### In silico gene structure prediction

The *Rwt3* NLR candidate gene transcript is 5,937 bp. Only one of the 15 annotated exons (grey-coloured exon in Fig. 2b) appears to be translated into protein. This exon encodes a protein of 1,069 amino acids with a coiled-coil (CC) domain, a nucleotide-binding (NB-ARC) domain and several leucine-rich repeats (LRRs) motifs at the C-terminus (Supplementary Fig. 4). The *Rwt4* NLR candidate gene has three exons encoding 1,038 amino acids with domains having homology to a CC domain, two NB-ARC domains and two LRRs at the C-terminus (Supplementary Fig. 5). The *Rwt4* WTK candidate has an open reading frame of 2,688 bp that has 12 predicted exons that encode a protein of 895 amino acids with putative tandem protein kinase domains (Fig. 2g and Supplementary Fig. 13). Domains were predicted by the National Center for Biotechnology Information (NCBI) and Pfam databases. The gene structure of both *Rwt3* and *Rwt4* NLR candidate genes was consistent with that predicted using complementary DNA RenSeq data of Watkins lines.

### Identification and phenotyping of Cadenza TILLING mutants to test the function of *Rwt4*

Cadenza TILLING lines^19^ for the NLR candidate for *Rwt4* were identified within the wheat Ensembl database (https://plants.ensembl.org/Triticum_aestivum/) for the gene TraesCS1D02G059000. Lines containing mutations leading to premature stop codons and those for which the ‘sorting intolerant from tolerant’ (SIFT) score was 0.0 or 0.01 were selected for phenotyping. For the *Rwt4* kinase candidate gene, Cadenza TILLING lines were identified for the gene TraesCS1D02G058900. Details of the mutations present in the Cadenza TILLING lines are provided in Supplementary Table 6.

### Identification and phenotyping of Jagger TILLING mutants to test the function of *Rwt3*

For selecting mutations in the *Rwt3* candidate gene (TraesCS1D02G029900), TILLING was performed in wheat cultivar Jagger^18^ using genome-specific primer pairs (Supplementary Fig. 10). The effects of the mutations on the predicted protein were analysed using SnapGene software (version 5.0.7 from GSL Biotech). The effects of mis-sense mutations were determined using Protein Variation Effect Analyzer (PROVEAN) v1.1 software^36^. Selected lines were phenotyped as described above. Details of the mutations are provided in Supplementary Table 6.

### KASP analysis and sequencing of TILLING lines to confirm mutations

KASP (LGC Genomics) was performed to confirm mutations where suitable PCR primers could be designed. Alternatively, the region containing the mutation was amplified and purified products were sequenced by Eurofins Genomics. Sequence analysis was performed with Geneious Prime software.

### VIGS using the BSMV

A 400 bp fragment of *Rwt3* and *Rwt4* (Supplementary Figs. 12 and 16) was cloned into the BSMV vector pCa-cbLIC via ligation independent cloning as described previously^37^.

### KASP marker design to detect *Rwt3* and *Rwt4* in wheat cultivars and Watkins collection

The regions differentiating *Rwt3* and *Rwt4* from their alternate allelic variants identified in *Ae. tauschii* and wheat were used to design KASP markers (Supplementary Table 7). KASP markers were validated on the sequenced Watkins panel of 300 lines (Supplementary Table 8), following which KASP marker analysis was performed on the entire Watkins panel (~900) to understand the distribution of these genes in the landrace collection (Supplementary Table 9).

### Characterization of the resistance identified on chromosome 7A

A set of Watkins lines were genotyped as carrying either *Rwt3* or the 7A resistance or having neither or both resistances. All lines were phenotyped in leaf assays using isolates Br48 and Br48 + *PWT3*. Lines lacking either resistance were susceptible to both isolates (Supplementary Fig. 17). Lines carrying either the 7A resistance alone or both the 7A resistance and *Rwt3* showed a similar level of resistance to both Br48 and Br48 + *PWT3*.

### Reporting summary

Further information on research design is available in the Nature Portfolio Reporting Summary linked to this article.

## Supplementary information

Supplementary Information Supplementary Figs. 1–18.

Reporting Summary

Supplementary Table Supplementary Tables 1–9; there are nine tabs in this workbook for each table.

Supplementary Data 1 Sequences for the new bait library.

Supplementary Data 2 Rwt3 (exon1) alleles multiple sequence alignment.

Supplementary Data 3 Rwt4 NLR alleles multiple sequence alignment.

## Data Availability

The RenSeq 150 bp paired-end Illumina sequences (raw data) for the 300 Watkins and 21 non-Watkins lines (including Anahuac) and the cDNA RenSeq data of 6 Watkins lines are available from NCBI study number PRJNA760793. The *k*-mer matrix and the CLC assemblies of the 300 Watkins and 21 non-Watkins lines are available from Zenodo under the following DOIs: 10.5281/zenodo.5557564, 10.5281/zenodo.5557685, 10.5281/zenodo.5557721, 10.5281/zenodo.5557827, 10.5281/zenodo.5557838 and 10.5281/zenodo.5655720. The genomic sequences of *Rwt3* and *Rwt4* are available from the wheat Ensembl databases as *TraesCS1D02G029900* from https://plants.ensembl.org/Triticum_aestivum/ and *TraesJAG1D03G00423590* from https://plants.ensembl.org/Triticum_aestivum_jagger/, respectively.

## Code availability

Scripts for the Watkins *k*-mer matrix generation, phylogenetic tree construction and *k*-mer-based association mapping can be found in the repository https://github.com/arorasanu/watkins_renseq.
