# Supplementary material for: A wheat kinase and immune receptor form host-specificity barriers against the blast fungus
Source: Nat Plants. 2023 Feb 16;9(3):385–92. doi: 10.1038/s41477-023-01357-5 (PMC10027608; doi:10.1038/s41477-023-01357-5)
Supplement: Supplementary file 1 — Supplementary Figs. 1–18. [file 41477_2023_1357_MOESM1_ESM.pdf]

nature plants

Brief Communication

https://doi.org/10.1038/s41477-023-01357-5

A wheat kinase and immune receptor form host-specificity barriers against the blast fungus

In the format provided by the authors and unedited

Supplementary Figures

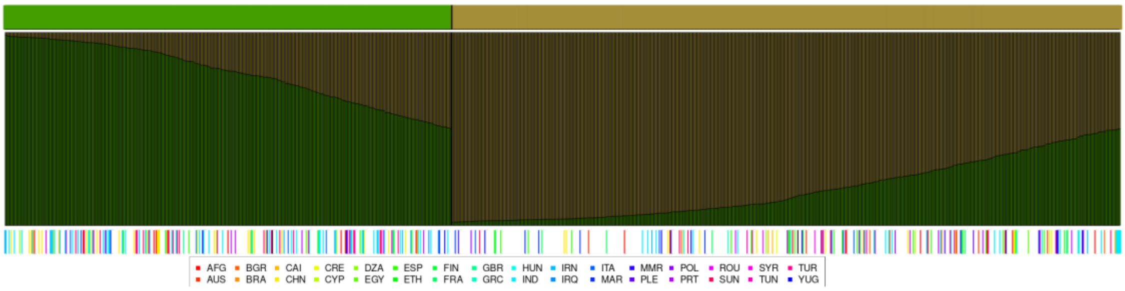

S1 STRUCTURE assignment of the landraces in the Watkins collection (1054 landraces) from Wingen et al (2014). The top row shows the assignment to a top-level split into two large ancestral populations. The middle row indicates the proportion of ancestral characteristics of each landrace cultivar (LCs). The bottom row colour code of country/region of origin is only shown for the 314 members of the core set. Unselected LCs are shown in white. Colour codes group countries into geographic regions.

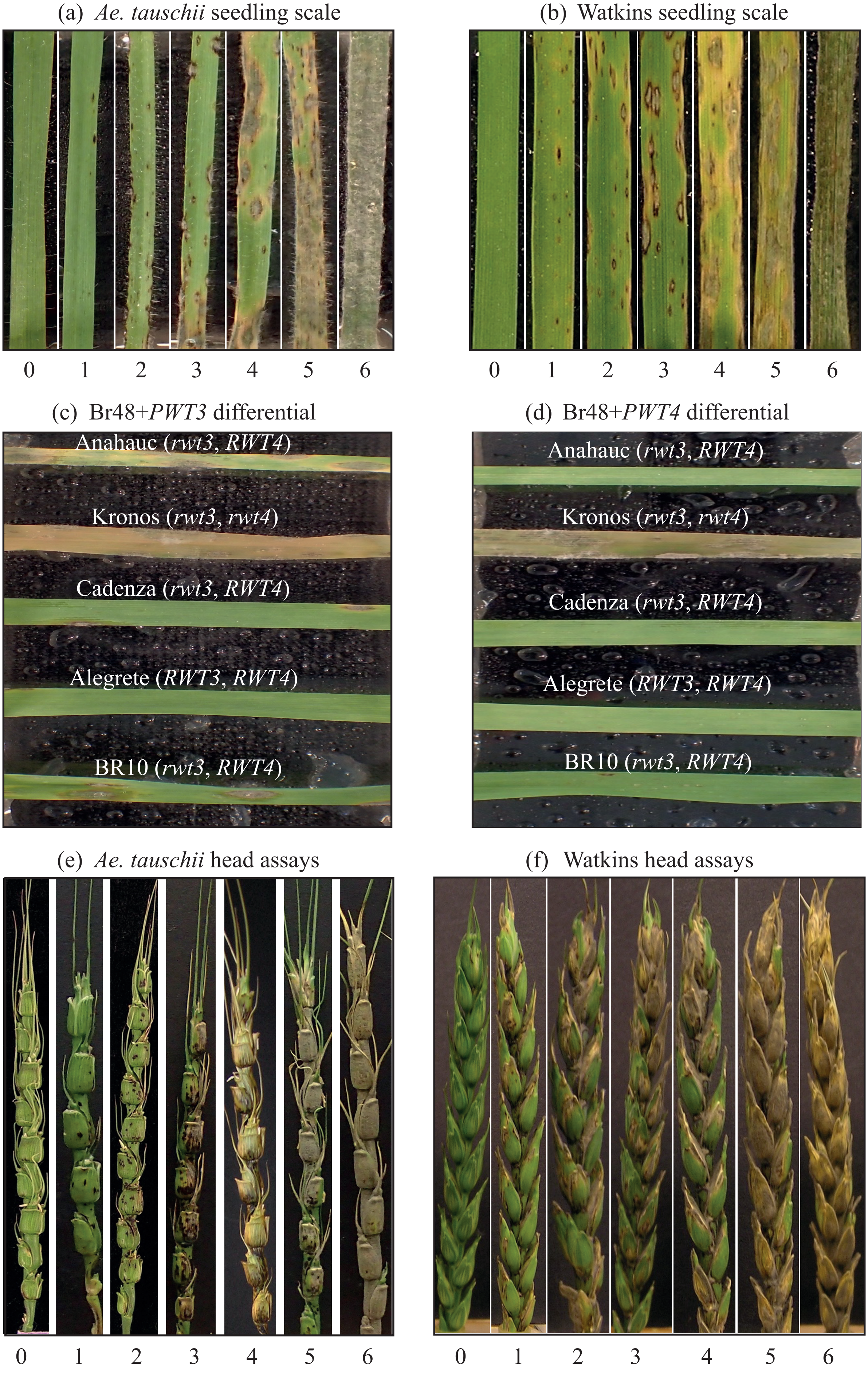

S2: Wheat blast detached leaves scoring scale (0-6) at 6 dpi for (a) *Ae. tauschii* and (b) Watkins panel. Variation observed for the differential lines (Anahuac, Kronos, Cadenza, Alegrete, BR10) upon phenotyping with (c) Br48+*PWT3* and (d) Br48+*PWT4*. Phenotype scale (0-6) at head stage for (e) *Ae. tauschii* and (f) Watkins panels.

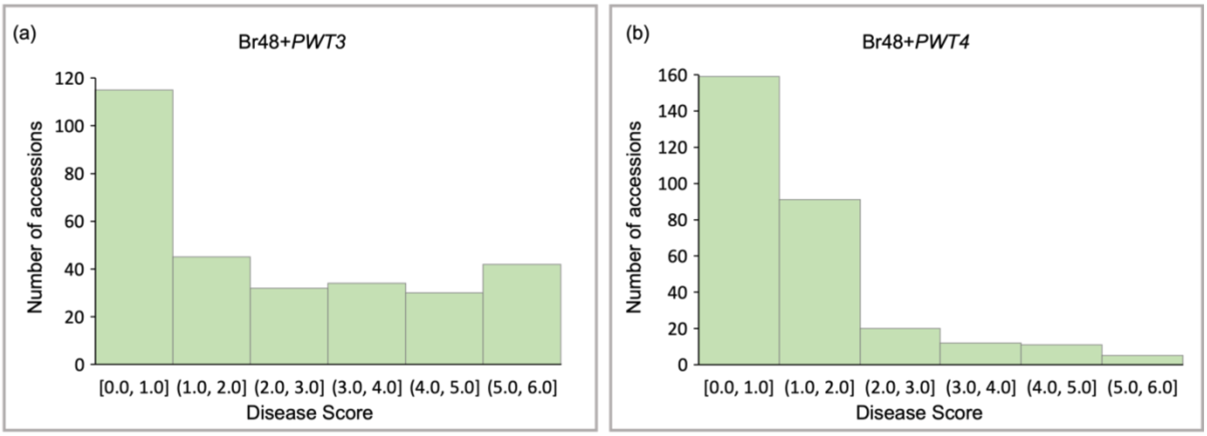

S3 Bar graphs showing phenotypic variation observed in the Watkins panel for (a) Br48+*PWT3* and (b) Br48+*PWT4*.

(a)

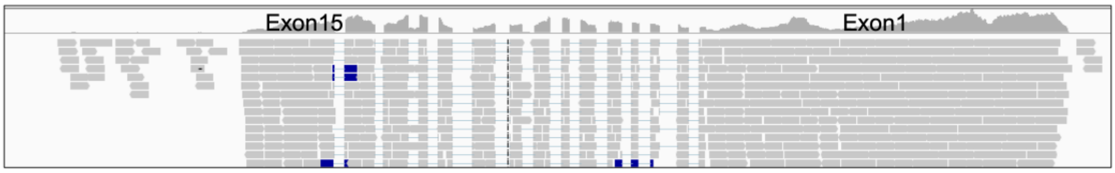

(b)
```
MAAALGAAQLLLGKVLTKLSDERMASYVASSELGLDSQKIKDDIMYTVGLLQQAQGRGTSDIPGLTDLLVKLSQKADEAEK
VLNELRYFMIRDQINGTQLVKPDLGACLKTKKGHARHTIGNCLPFIFRPCTSSQQDGTMSDGHDHVDELPFDSVSISIKIK
SVLEETHSICVHVSDLLKLIPNHGSSSTTATTVTRIRPTRGSMVAQDTMYGRRDIFEKTIHAITSCGEILSVLPVVGPGGI
GKTTLIQHLYNDKRIEEHFDVRIWVCVSIDFAVLKLTREIFRCIIATEEEGRNSAPSETASIEQLQESIAQRLQSKRFLIV
LDDVWQLNSEDEWHTLLAPFKKGGAKGNMVLVTTRFPSIAGRVKTVDPVELQGLDTDDFFAFFEECIFGGLDKPMHYRDEL
TDIARDIVDKLKGSPLAAKTVGRLLRKDLSWEHWTRVLESMEWENKQSDNDTMPALYLSYDYLPFLLQKCFSYLSLFPEDH
RFSHSEINRFWIAVGIIDSNHPGNNNCLEELVDRGFLRRVHGLFGEEYYVIHDLLHELSRSVSTQECLNISVLDFKADTVP
PSIQHLSITLENKYDGIFLEEISKLKIKIDIANLRTLMIFSAYEERIAGILKDTFEEVDSLHVLFIVVKSLDDLPKGFSKL
IHLQYLKLGSPIGIEMALPSTLARFYHLKFLDLKDWHGSSNVPKDISHLVNLQDFIAKKELHSSVLEVGKMKYLRELKQFC
VKKESVGFELRELGELTELGGELRICNLENVATKEEASEAKLMSKRNLKKLTLVWGRKQSTIDGDVLDALQPHPDLRELRI
ANHGGAVGPSWLCVDMLLKQLGVLHLEGLSWDTLPPFGQLPRLTKLILMRISGVHQLALLSNLTSLTHLTLQDCVKLTVDG
FNPLTTVNLKVLVVFNCRWDRSCPESVAADLLTKVASSRVMPAGSFRLEQLKVDSISAVLVTPICNLVAHTLQTLIFCHDH
RIKSFTEEQEKALLLLTSLRHLTFDGCGALQSLPRGLNRLSSLKDLEVLWCPEMGSIPKEGFPVSLEILRIRPCSPEVREQ
IEKLRRTSPGLSVRYE
```

S4 (a) Mapping of RNA-Seq reads to the *Rwt3* NLR candidate gene present in the Chinese Spring genome and (b) the predicted amino acid sequence of the *Rwt3* gene with coiled-coil (orange), NB-ARC (yellow) and LRR (blue) domains.

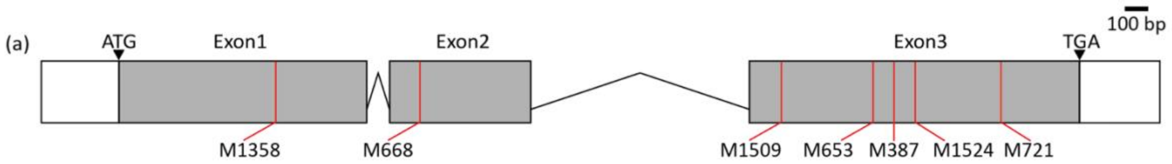

(b)

MEEIVLGLSKTVVEGTLVKVKAAIDEEAKLKLAVQSDLVFITGEFEMMQSFLNVADAERIKNNAVKTWVRQLRDLAYDTEDCIELVVHLDPKPR
WWRRLLVLPCLPAVSLPMDNAAAEIKELKDRVEFVSQRNMRYKLITDFGGAKSVAQQQLDRAAGALDIAVEEAAKKGRSLVDLTELIPRMEDRP
ELGVISVWGTGGDLGVASIVRKAYDDPEICKNFHSRGWAKLTHPFDPRKILRSLFIQFCTNTAAPQKQGETLDVDSLLRMEKTVVEEGELVKQF
VSHVSTHRFLVVLEGVSTMAEWDALRMYLPDMGNGSQIIVSTQHFDIASLCTGQLHKVSELRKFTPDHSVCVFFKEVSGGDDPGNAMLEGDDET
ELIGRDTEEDELMEQIDEVSRVISVWGIAGVGKSALVRSAYCKYLQDRPNFAGLNLTYIHGRVNVPHPFNLREFCRSLLLNLSSEPAKTKEDAV
AELANMTNPIDVCRKLLSDDQQQKQCLIVIDDLQSTEEWDRIKQALSFGKECCIIVVTNDSSVAKYCSGQDQKLMFNVKGLGDEHALDLFNTVY
DGVGNHITRDEDMINQSKLMLNKCGGLPKVIVAIGCFLAKSLNWEAMNTNFIHQLENNPELASVHSMFSWLDSYFHNCPDELKPCIFYLSIFPR
RHGIRRRRLVRRWIAEGYSRDTDGNLAEVNGENYFSRLVNLSMLTDAERDVNVAGTATGAGTGRRMAMCNVNDFFREYIVSRRMEENHVFALEG
RCSQTTRRTGRHLVIDKSWDGDESVFNRIEFSRLRSVTVFGPWKPFLASDKMRVLRVLDLEGTEGLTDNDIKNIVKRLPRLKFLSLRGCKHIFR
LPKSLGRLRQLQTLDVRHTAVAGLPATVVKLQKLQYVRAGNTPEEYAVSDSTESSTSVPTCFSCGPVGNHIVGVEVPRGVENLTNLHTLGVIKA
TVAGLKELKKLTQLRKLGVSGINRRNHKELCAVISGHGHLESLSIWFDKDTERIADPCSPSNDDFKPPEKLRRLKLHGHTDNLPAWVSTLKIFV
TQDQ

S5 The intron-exon structure of the NLR candidate gene for *Rwt4*. The predicted 1038 amino acid protein has domains with homology to a coiled-coil (orange), nucleotide-binding (yellow) and leucine-rich repeats (blue).

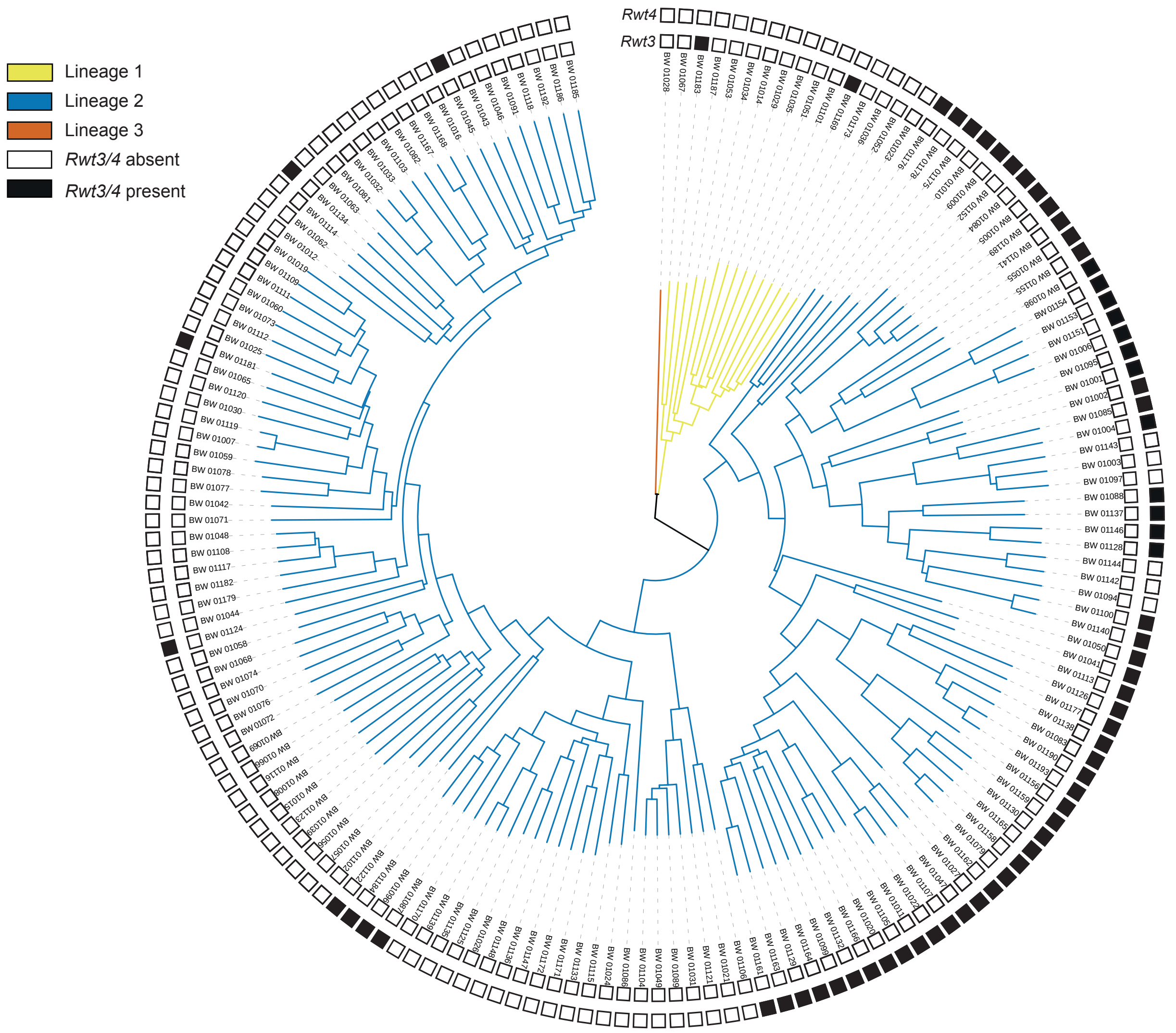

S6 Presence of *Rwt3* and *Rwt4* candidate resistance genes in the phylogeny of the *Ae. tauschii* panel (adapted from Arora *et al* 2019).

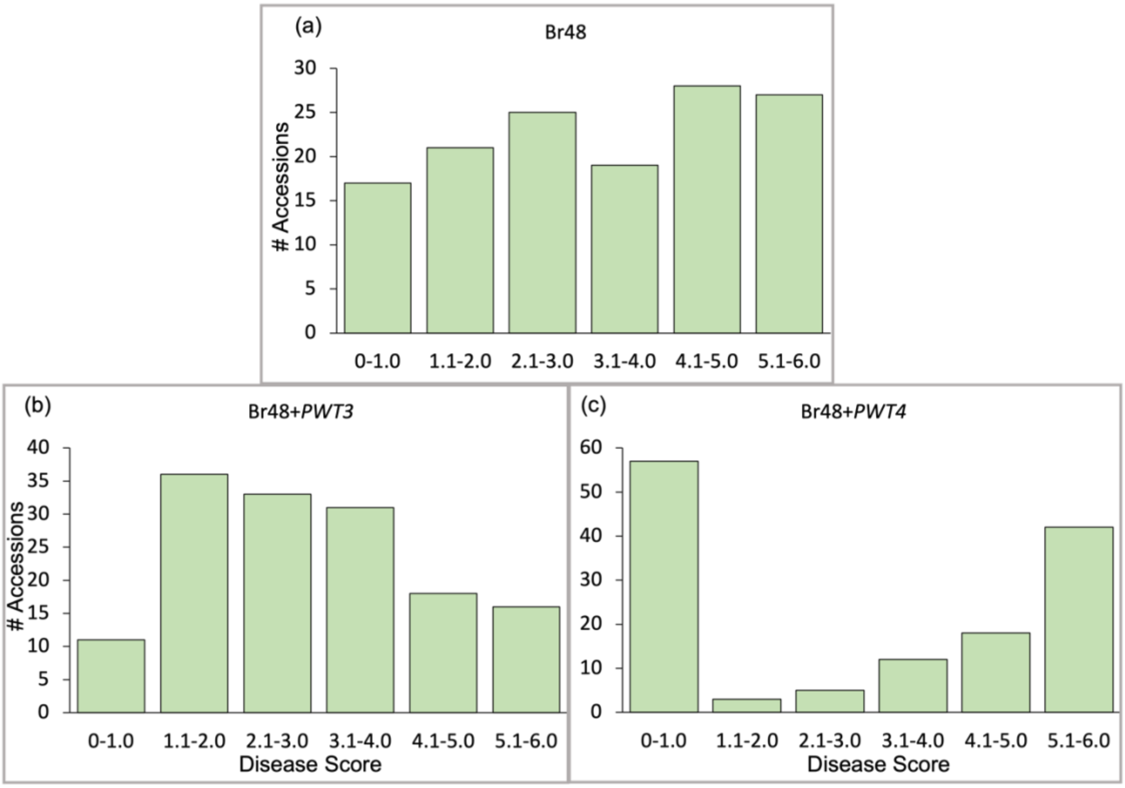

S7 Bar graphs showing phenotypic variation observed in the *Ae. tauschii* panel for (a) Br48 (b) Br48+*PWT3* and (c) Br48+*PWT4*.

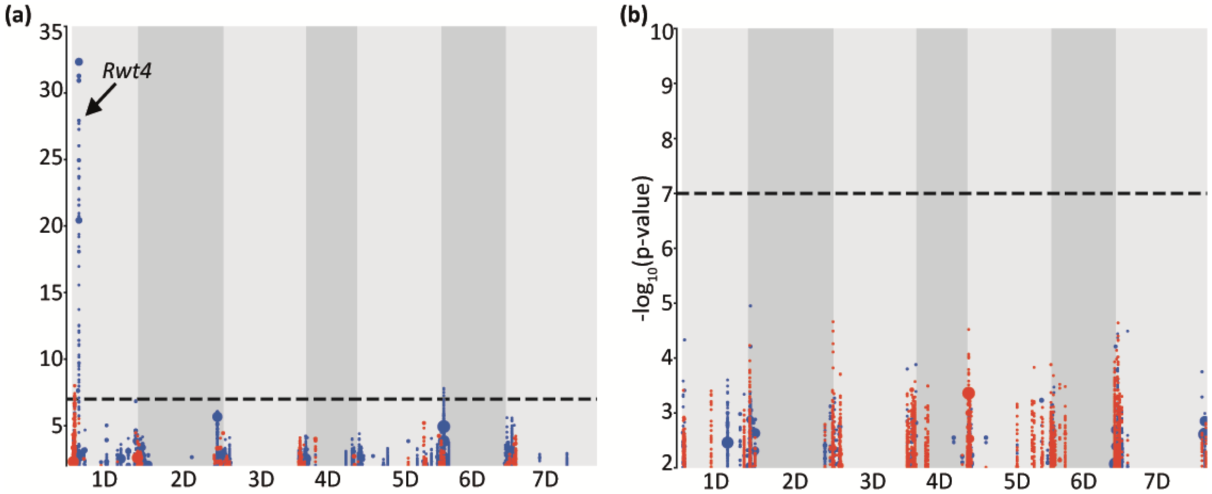

S8 Association mapping plots showing (a) identification of a clear peak in the *Ae. tauschii* panel screened with Br48+*PWT4*, however, (b) no clear association was observed for *PWT3* recognition in this panel. Points on the y-axis depict *k*-mers positively associated with resistance in blue and negatively associated with resistance in red. Point size is proportional to the number of *k*-mers. The association score is defined as the $-log_{10}$ of the *p*-value obtained using the likelihood ratio test for nested models. The threshold of significant association scores is adjusted for multiple comparisons using the Bonferroni approximation.

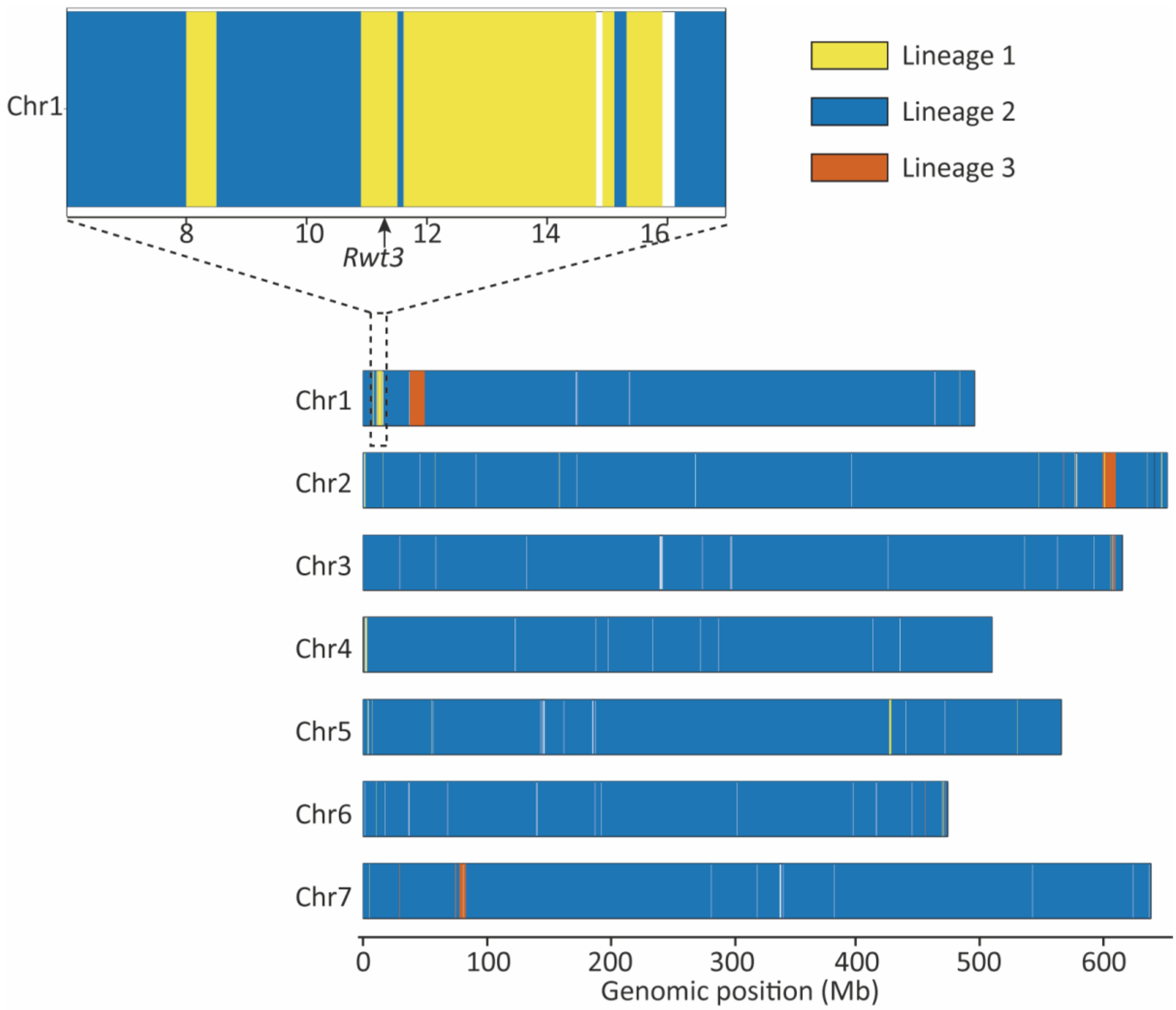

S9 The pattern of lineage-specific contribution to the Chinese Spring D-subgenome across all the seven chromosomes (adapted from Gaurav et al 2021). The genomic region most enriched with lineage 1 contribution is located on chromosome 1DS surrounding *Rwt3*.

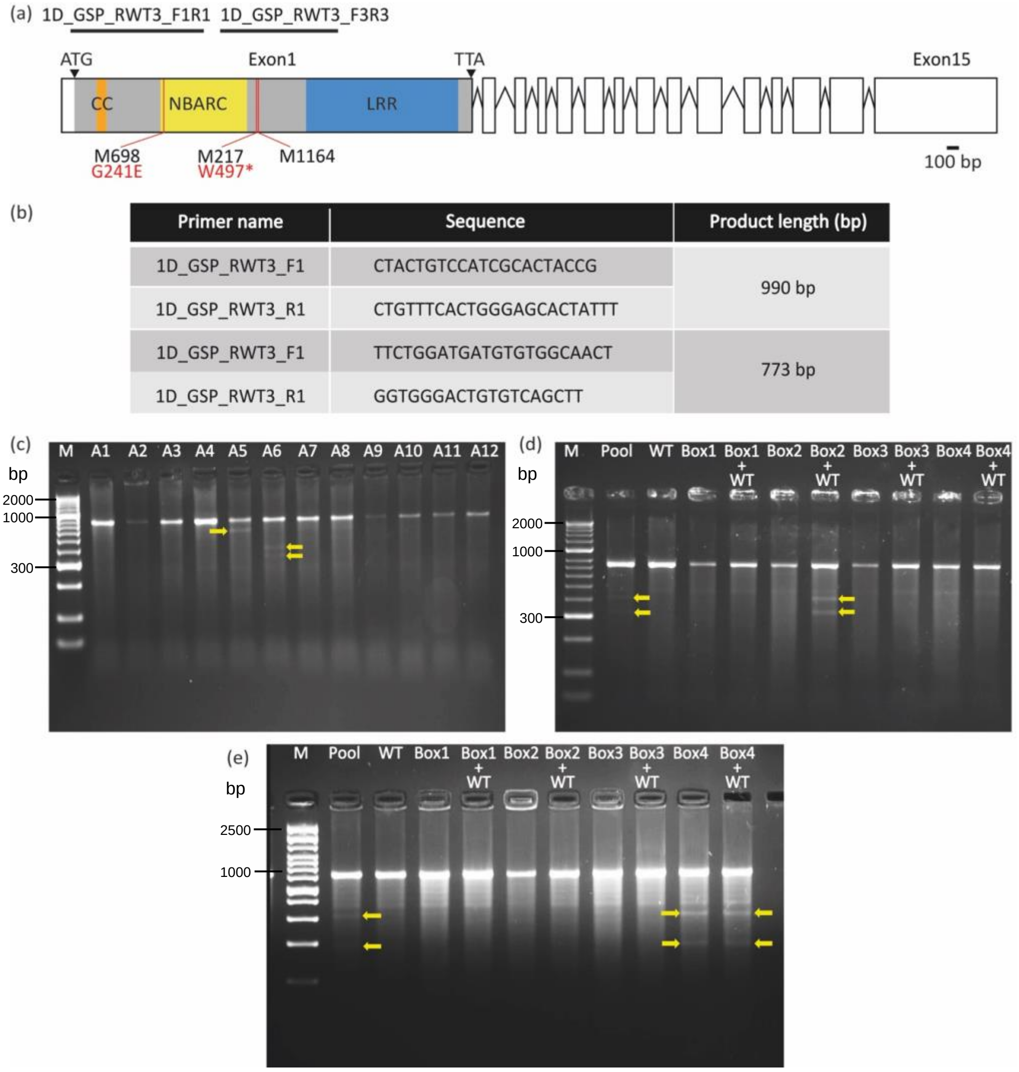

S10 *Rwt3* Jagger mutant selection (a) Position of the two primer pairs in the *Rwt3* gene selected for performing TILLING and location of the mutants on the gene selected for disease phenotyping. (b) Primers used for TILLING the Jagger mutagenized population. (c) Identification of mutants in a row of 12 pools of the Jagger TILLING population, Deconvolution and zygosity determination of: (d) a homozygous mutant and (e) a heterozygous mutant. TILLING Results were replicated at least twice before confirming mutations via Sanger sequencing.

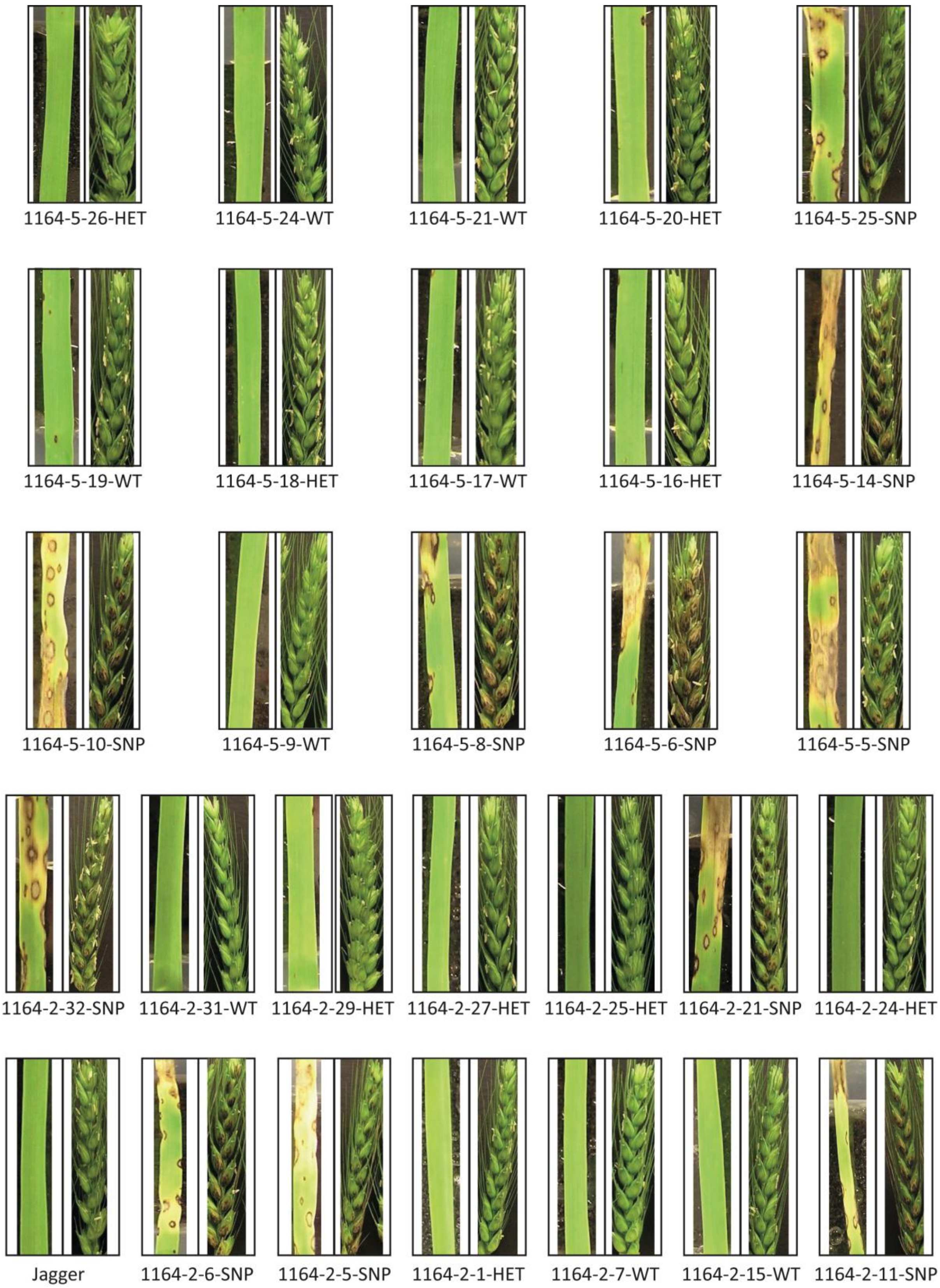

S11 Leaf and head assays of the segregating progeny of Jagger for *Rwt3* heterozygous mutant 1164 using Br48 + *PWT3*.

ATGGCGGCGGCTCTTGGTGCGGCGCAGTTGCTCCTCGGCAAGGTGCTCACGAAGCTCTCGGATGAGCGTATGGCC
TCGTATGTGGCTAGCTCCGAGCTTGGCCTCGACTCCCAGAAGATCAAAGATGACATTATGTACACGGTTGGCCTG
CTGCAGCAGGCCCAGGGGAGGGGCACCAGCGACATTCCTGGCCTCACGGACTTGCTGGTGAAGCTAAGCCAGAAG
GCCGATGAGGCTGAGAAAGTGCTGAACGAGCTCCGGTACTTCATGATCCGGGACCAGATTAACGGCACCCAGCTA
GTCAAGCCAGATCTGGGCGCTTGCCTCAAGACCAAGAAAGGTCATGCTCGTCACACTATTGGTAACTGTCTGCCA
TTCATTTTTCGTCCATGTACCAGTAGCCAACAAGATGGAACCATGTCTGATGGCCATGACCATGTTGATGAGTTG
CCATTTGATAGTGTGTCCATATCCATCAAAATCAAGTCGGTGTTAGAGGAGACACACTCCATATGTGTTCATGTC
TCCGACTTGCTCAAGTTGATTCCAAACCACGGCAGCAGCTCTACGACAGCCACAACTGTCACCCGCATACGGCCT
ACTAGAGGATCAATGGTTGCACAGGATACAATGTATGGTAGGCGGGACATCTTTGAGAAAACTATACATGCCATC
ACCAGTTGCGGCGAAATCCTTTCTGTTCTCCCTGTAGTTGGGCCAGGGGGCATTGGAAAAACAACTTTAATCCAA
CACCTGTATAATGATAAAAGGATTGAAGAACACTTTGATGTCAGAATATGGGTATGTGTATCAATTGATTTTGCT
GTGCTTAAGCTCACCCGGGAGATCTTTAGATGCATAATTGCAACTGAAGAAGAAGGAAGAAATAGTGCTCCCAGT
GAAACAGCCAGTATAGAACAGCTTCAGGAGTCTATTGCACAGAGGCTCCAGTCCAAAAGGTTTTTAATTGTTCTG
GATGATGTGTGGCAACTCAATAGTGAAGATGAGTGGCATACCCTATTAGCTCCATTCAAAAAGGGAGGAGCCAAG
GGCAACATGGTACTTGTCACAACCCGGTTCCCATCTATAGCAGGCAGGGTGAAGACAGTTGATCCAGTCGAGCTG
CAAGGTTTAGACACTGATGATTTCTTCGCATTCTTTGAAGAGTGTATATTTGGTGGACTCGATAAGCCTATGCAT
TACAGAGATGAGTTAACTGATATTGCAAGAGATATTGTAGATAAGTTAAAGGGTTCACCTTTAGCAGCCAAAACA
GTCGGCCGCCTATTAAGGAAAGACCTATCCTGGGAACACTGGACAAGAGTTCTTGAAAGCATGGAATGGGAAAAT
AAGCAGAGCGATAATGATACTATGCCAGCTTTGTACCTTAGCTACGATTACCTCCCTTTCCTCCTGCAAAAATGT
TTTTCCTATCTTTCTCTTTTTCCTGAAGATCATAGGTTTAGTCATTCAGAAATTAATCGCTTTTGGATTGCAGTA
GGCATCATAGACTCTAATCATCCAGGAAATAACAATTGCTTGGAAGAACTAGTGGACCGTGGTTTTCTTAGGAGG
GTACATGGTTTGTTTGGTGAAGAATACTATGTAATCCATGATTTACTGCATGAACTATCTCGAAGTGTTTCAACA
CAAGAATGCCTCAATATAAGTGTTTTAGATTTTAAAGCTGACACAGTCCCACCATCTATTCAACACTTATCTATC
ACCCTAGAAAATAAATATGATGGAATTTTTTTGGAAGAAATAAGTAAACTGAAAATAAAGATAGACATTGCAAAT
TTACGGACTTTAATGATTTTTAGTGCATATGAAGAAAGAATTGCTGGTATTTTAAAAGATACGTTTGAGGAAGTA
GATAGTCTGCATGTCCTATTTATAGTTGTGAAATCCCTGGATGATTTGCCAAAAGGCTTTTCAAAACTTATCCAC
CTCCAGTACCTCAAACTTGGATCACCTATTGGCATAGAAATGGCATTACCTAGCACACTGGCCAGATTTTATCAC
TTGAAATTCTTAGACCTAAAAGATTGGCATGGTAGTTCTAATGTTCCTAAAGACATTAGTCACCTTGTGAATTTG
CAAGACTTCATTGCTAAAAAAGAACTCCACTCCAGTGTTCTTGAGGTTGGAAAGATGAAGTACCTACGGGAACTA
AAACAATTCTGTGTTAAGAAAGAGAGTGTTGGGTTTGAATTAAGAGAGCTAGGGGAACTGACAGAGCTTGGGGGA
GAACTCAGAATATGTAACCTTGAAAACGTGGCAACCAAGGAAGAAGCTAGTGAGGCCAAACTGATGTCGAAAAGG
AATCTGAAGAAGCTGACATTGGTTTGGGGCAGAAAACAATCGACTATAGATGGTGATGTTCTTGATGCTCTTCAA
CCGCATCCTGATCTTAGAGAGCTTCGAATTGCAAATCATGGTGGTGCTGTTGGTCCCAGTTGGTTGTGTGTTGAC
ATGTTGCTGAAACAGTTAGGTGTTCTCCATCTCGAAGGTTTGTCTTGGGACACTCTTCCACCTTTTGGGCAGCTA
CCACGCCTCACTAAACTCATTTTGATGAGGATTTCTGGAGTGCATCAGTTGGCTCTGCTCTCAAACCTCACGTCT
CTCACCCATCTGACACTGCAAGATTGTGTCAAGTTAACAGTGGATGGATTCAATCCTCTTACCACAGTCAACCTT
AAGGTATTGGTGGTATTTAACTGCCGATGGGACAGAAGTTGTCCCGAATCTGTAGCAGCGGATCTTCTCACAAAG
GTGGCAAGTAGCAGAGTAATGCCTGCAGGTTCCTTCCGATTAGAACAGCTCAAGGTGGACAGCATCTCGGCAGTG
CTTGTCACTCCCATCTGCAACCTTGTCGCCCATACCCTCCAGACTCTGATATTCTGCCATGATCACCGGATCAAG
AGTTTCACAGAAGAGCAAGAGAAGGCGCTTCTGCTCCTCACATCACTCCGACACCTCACATTTGATGGTTGTGGG
GCTCTGCAGTCCCTCCCGAGAGGGTTGAATCGCCTTTCTTCACTCAAAGACTTAGAGGTCCTCTGGTGCCCTGAA
ATGGGATCCATACCCAAGGAGGGGTTCCCAGTTTCGCTCGAAATCCTACGCATAAGACCTTGCAGCCCCGAGGTT
AGGGAGCAAATTGAGAAACTCAGAAGAACAAGCCCAGGTTTATCTGTACGATACGAGTAA

S12 *Rwt3* sequence with region used for generation of Virus Induced Gene Silencing construct highlighted and primers used to generate amplicons underlined.

```
MGGYEFQRAELDALEGVVRDPTAEPMSLTLPLLRHITNDFSPEFEISKDDSAVVYLGVLPSGFRVAVKKSHFRFCLDDEDAFT
NEVSIAMKAAHKNTVRVIGYCHHTHEQIAEYEGKQVFAEVRERLICTEYVPNGPLSGHIEGKICAQMDGYEFQRAELDALERV
VRDTSAEPMSLTLPLLRHITNDFSDESRIGRGGFAVVYLGVLPSGLRIAVKRLSNIAYMNESAFQNEVFITMKATHKNTVRFM
GYCSQIQGKLIEHDGQHVFAQLEERLICVEYAPKGTLDAHIGDYGELDWNQRYQILKGICQGLHHLHDEMHVFHGDIKPANIL
IGDNLVPKIYDFGLSQMFEEEETERIVENIAGTFGYMAPEFCTNNMVSFKAEIYSLGVVIGELLIGKKGWFDEDVRKLFVQQL
KGLRKTLVKEGAFSSWENKYHQVRTCMEIGQDCIDPNPHKRPTLLEIIQRLNEAEDMNYSAASLWQSGDEESDLSDTEALETE
TTSEFLPSDEEPASVGKTGETSTQEPDKPDLISKLPASVDLSDLKVLEKITDDFSHERIVGKDGTFKGCHKAFVYKGDIPLRE
MIAVKRLIGVEIPFEKFKREAEQFISLDHKNIVKVASYCHDQSRGHRLVQFKGKPLPQLFNGPEQLLCYEYMHNGSLRDYLMG
QGSRVIDWQMRYKLIKGTCAGLHYLHKGRAGCPIVHLNLSPSNVLLDHNYIPRITGFDFSKLIGEKNTKSVVLKLNGPIAYLP
PDFFHSKGTDLKYLATVDIYSLGLMILEIATQQEIKGIHGVLIKSIEENWREESQITRLYTSLGADELRQVKMCIDIGLDCVK
SNPEKRPTAGAIMLWLDKESKPVPVSRAGAGVLPRPPVPTNINHAGRIQEKEKAGFLKRHFGWKK
```

S13 Protein sequence of the *Rwt4* WTK gene with two (green and orange coloured) predicted kinase domains.

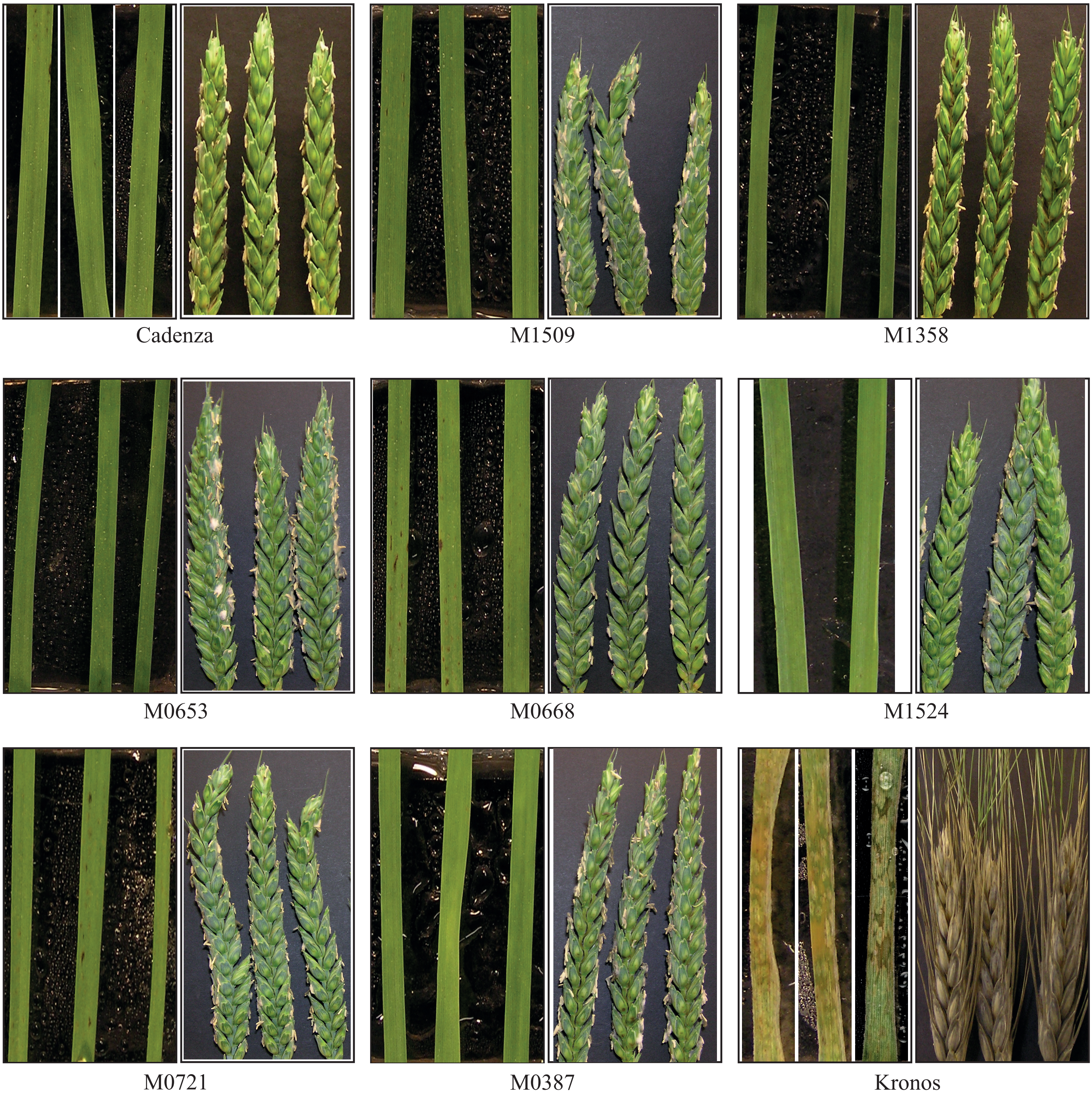

S14 Phenotyping of the mutants for the *Rwt4* NLR candidate gene with Br48+*PWT4* at both seedling and head stage.

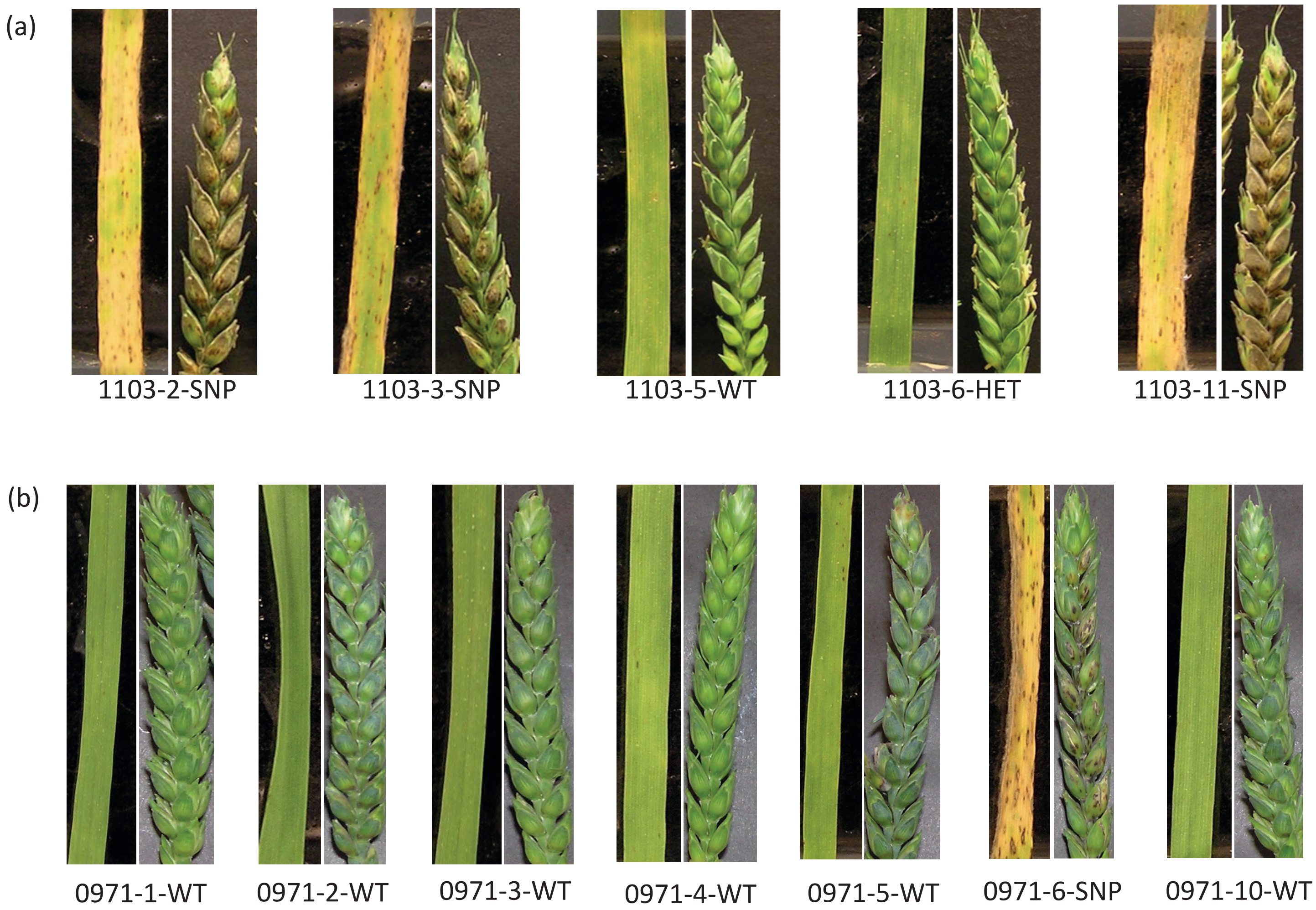

S15 Genotype and phenotype correlation of the segregating progenies of *Rwt4* WTK mutants (a) Cadenza 1103 and (b) Cadenza 0971.

ATGGGCGGATACGAGTTCCAGAGGGCGGAGCTAGATGCACTGGAAGGCGTCGTACGCGATCCAACTGCGGAGCCA
ATGAGTCTGACGTTGCCGCTTCTCAGGCACATAACAAATGATTTCTCCCCTGAATTTGAAATTAGTAAAGATGAT
TCTGCAGTGGTTTACCTGGGGGTGCTTCCAAGTGGGTTCCGTGTTGCTGTCAAGAAGTCTCACTTTCGTTTTTGC
TTGGATGATGAAGATGCATTCACAAATGAAGTTTCTATTGCAATGAAGGCTGCTCATAAGAACACAGTGCGAGTC
ATAGGCTACTGTCATCACACGCATGAGCAAATTGCCGAATACGAAGGAAAACAAGTTTTCGCAGAGGTCAGAGAA
AGGTTGATCTGTACCGAGTATGTGCCTAACGGACCCCTTAGTGGACATATCGAAGGTAAGATATGTGCGCAAATG
GATGGATACGAGTTCCAGAGGGCAGAACTAGATGCACTAGAACGCGTCGTACGCGATACAAGTGCGGAGCCAATG
AGTCTGACGTTGCCGCTTCTCAGGCACATAACAAATGATTTCTCCGATGAATCTCGAATTGGCCGAGGTGGATTC
GCAGTGGTTTACCTGGGGGTGCTTCCAAGTGGGTTACGTATTGCTGTTAAGAGGCTTAGCAATATTGCTTATATG
AACGAAAGTGCATTTCAAAATGAAGTGTTTATCACAATGAAGGCCACTCACAAGAACACAGTGCGATTCATGGGC
TACTGTAGTCAAATACAAGGTAAACTCATCGAACACGACGGCAACATGTTTTCGCACAGCTCGAGGAAAGGTTG
ATCTGTGTGGAATATGCGCCTAAAGGAACCCTTGATGCACATATCGGTGACTATGGTGAACTTGACTGGAACCAG
CGTTATCAAATTCTAAAAGGAATTTGTCAAGGTTTGCATCATCTCCATGACGAAATGCACGTTTTTCATGGAGAT
ATCAAACCAGCCAATATATTAATAGGGGATAACCTTGTGCCTAAAATCTATGACTTCGGTCTCTCCCAGATGTTT
GAAGAAGAAGAAACGGAACGTATTGTTGAAAATATCGCCGGAACATTCGGATATATGGCACCGGAGTTTTGTACT
AATAATATGGTGTCATTTAAGGCTGAGATATACAGTTTGGGCGTTGTGATCGGGGAGTTATTGATCGGGAAGAAA
GGATGGTTTGATGAGGATGTGAGAAAACTATTTGTACAGCAACTTAAGGGTTTGAGAAAAACATTGGTAAAAGAA
GGAGCGTTTTCATCATGGGAAAACAAATACCACCAAGTTAGAACATGTATGGAGATTGGGCAGGACTGCATAGAC
CCCAACCCACATAAAAGGCCCACTTTGTTGGAGATTATCCAGCGGCTTAATGAAGCGGAAGATATGAACTATTCT
GCAGCATCACTTTGGCAGTCAGGAGACGAGGAATCCGATTTATCGGATACAGAAGCTTTGGAGACAGAGACAACA
TCCGAGTTTCTTCCAAGTGACGAAGAACCCGCCTCTGTGGGCAAGACCGGAGAAACAAGCACACAGGAGCCTGAT
AAACCGGACCTAATAAGTAAGTTGCCAGCATCGGTGGACCTGTCTGACCTAAAAGTCCTGGAGAAAATCACAGAT
GATTTTTCACACGAAAGAATAGTTGGGAAGGACGGTACATTCAAAGGTTGTCATAAGGCATTTGTTTATAAGGGT
GACATTCCACTTAGAGAAATGATAGCCGTGAAGAGGTTAATTGGAGTGGAGATTCCATTTGAAAAGTTTAAGAGG
GAAGCAGAACAGTTCATTAGTCTCGATCATAAGAATATAGTAAAGGTTGCCAGCTACTGCCACGACCAGTCTAGA
GGACATAGACTGGTACAGTTCAAAGGAAAACCGCTACCACAACTCTTTAACGGTCCCGAACAACTGCTCTGCTAT
GAATATATGCACAACGGAAGCCTTCGCGACTATCTTATGGGTCAAGGATCTCGTGTAATTGATTGGCAAATGCGC
TACAAATTGATCAAAGGGACTTGCGCAGGCTTACATTACCTTCACAAGGGCCGTGCAGGTTGTCCAATTGTTCAT
TTGAATTTAAGCCCGTCAAATGTATTGCTGGACCACAACTACATACCACGCATCACAGGGTTCGATTTTTCGAAG
CTCATTGGTGAAAAGAACACCAAATCAGTGGTACTTAAGCTGAATGGACCCATAGCGTACCTGCCACCGGATTTC
TTCCATTCGAAGGGTACTGATCTTAAATATCTTGCTACGGTAGATATATACAGCTTGGGTCTTATGATTTTAGAA
ATCGCAACACAACAAGAGATCAAAGGCATCCATGGAGTGCTTATTAAGAGTATAGAGGAAAACTGGAGGGAGGAG
TCACAAATAACACGGCTGTATACCTCACTAGGGGCCGACGAGCTGCGGCAAGTAAAAATGTGCATTGATATTGGC
CTAGACTGTGTCAAGTCAAACCCTGAAAAGAGACCTACAGCTGGGGCCATCATGCTCTGGCTTGACAAAGAGAGC
AAACCGGTCCCAGTTTCAAGGGCAGGTGCAGGAGTGCTGCCAAGACCTCCGGTCCCTACTAATATCAACCATGCA
GGTCGCATCCAAGAAAAGGAGAAGGCGGGATTCCTGAAACGACACTTCGGATGGAAGAAGTAA

S16 *Rwt4* sequence with region used for generation of Virus Induced Gene Silencing construct highlighted and primers used to generate amplicons underlined.

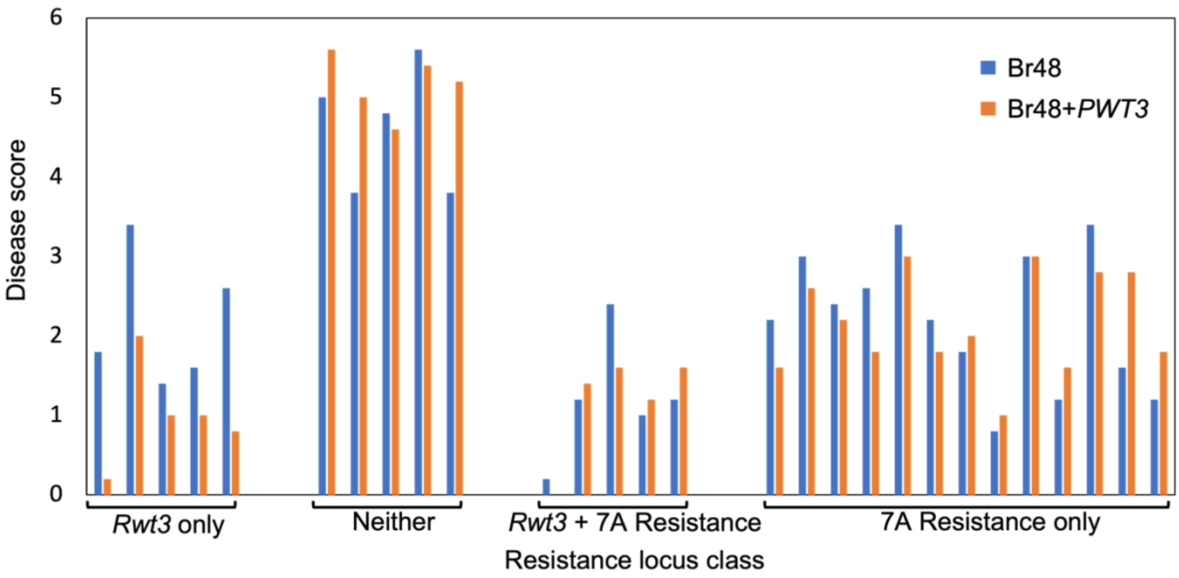

S17 Effect of the presence of *Rwt3* and the resistance locus identified on chromosome 7A on the resistance to Br48 and Br48+*PWT3*.

Future risk of regaining *PWT4* effector

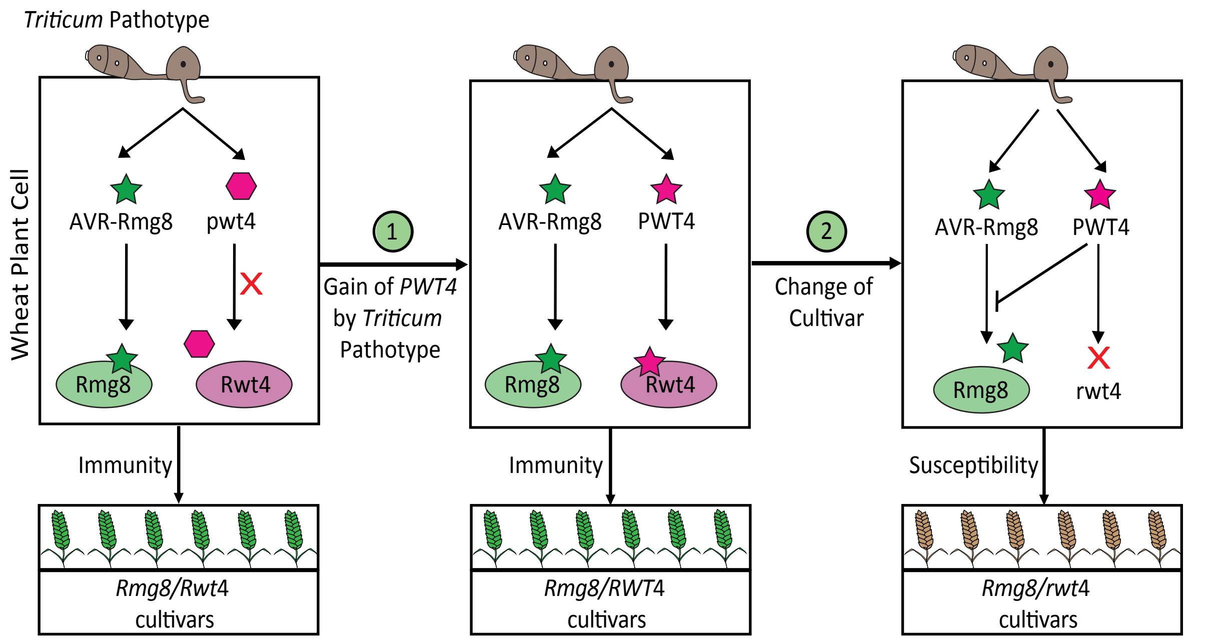

S18 A potential risk posed by the *Triticum* pathotype gaining *PWT4* effector in the absence of *Rwt4*.
(i) A schematically drawn wheat cell of a cultivar carrying *Rmg8* and *Rwt4* attacked by a *Triticum* isolate carrying *AVR-Rmg8*. The *AVR-Rmg8* effector isrecognized by *Rmg8*, thus preventing the *Triticum* isolate from infecting the cultivar.
(ii) A *Triticum* isolate that has gained the *PWT4* effector from non-*Triticum* pathotypes via sexual recombination is still not able to infect cultivars carrying *Rwt4*.
(iii) However, cultivars lacking *Rwt4* (or having the susceptible allele, *rwt4*) will be susceptible to the *Triticum* isolate carrying both *AVR-Rmg8* and *PWT4* even if the cultivar carries *Rmg8* because in the absence of *Rwt4*, *PWT4* suppresses the recognition of *AVR-Rmg8* by *Rmg8*.
