# Supplementary material for: A wheat kinase and immune receptor form host-specificity barriers against the blast fungus
Source: Nat Plants. 2023 Feb 16;9(3):385–92. doi: 10.1038/s41477-023-01357-5 (PMC10027608; doi:10.1038/s41477-023-01357-5)
Supplement: Supplementary file 2 — Reporting Summary [file 41477_2023_1357_MOESM2_ESM.pdf]

nature portfolio

Corresponding author(s): Paul Nicholson
Brande Wulff

Last updated by author(s): 03-1-2023

Reporting Summary

Nature Portfolio wishes to improve the reproducibility of the work that we publish. This form provides structure for consistency and transparency in reporting. For further information on Nature Portfolio policies, see our Editorial Policies and the Editorial Policy Checklist.

Statistics

For all statistical analyses, confirm that the following items are present in the figure legend, table legend, main text, or Methods section.

n/a	Confirmed	
☐	☒	The exact sample size (*n*) for each experimental group/condition, given as a discrete number and unit of measurement
☐	☒	A statement on whether measurements were taken from distinct samples or whether the same sample was measured repeatedly
☐	☒	The statistical test(s) used AND whether they are one- or two-sided<br>*Only common tests should be described solely by name; describe more complex techniques in the Methods section.*
☐	☒	A description of all covariates tested
☐	☒	A description of any assumptions or corrections, such as tests of normality and adjustment for multiple comparisons
☐	☒	A full description of the statistical parameters including central tendency (e.g. means) or other basic estimates (e.g. regression coefficient) AND variation (e.g. standard deviation) or associated estimates of uncertainty (e.g. confidence intervals)
☐	☒	For null hypothesis testing, the test statistic (e.g. *F*, *t*, *r*) with confidence intervals, effect sizes, degrees of freedom and *P* value noted<br>*Give P values as exact values whenever suitable.*
☒	☐	For Bayesian analysis, information on the choice of priors and Markov chain Monte Carlo settings
☒	☐	For hierarchical and complex designs, identification of the appropriate level for tests and full reporting of outcomes
☐	☒	Estimates of effect sizes (e.g. Cohen's *d*, Pearson's *r*), indicating how they were calculated

Our web collection on statistics for biologists contains articles on many of the points above.

Software and code

Policy information about availability of computer code

Data collection	No software was used during data collection.
Data analysis	Scripts for the Watkins k-mer matrix generation, phylogenetic tree construction and k-mer based association mapping can be found in the repository https://github.com/arorasanu/watkins_renseq.<br>The above custom scripts were written and tested in Python 3.6.13 alongwith following Python modules:<br>numpy (v1.17.0)<br>pandas (v0.23.0)<br>Biopython (v1.78)<br>scikit-learn (v0.24.2)<br>statsmodels (v0.12.2)<br>bitarray (v2.3.0)<br>matplotlib (v3.3.0)<br><br>Additionally, the following softwares were used for data analysis in the study:<br>BLAST+ command-line tools for alignment version 2.2.28<br>CD-HIT (v4.6.8)<br>BioMart (https://ensembl.org/info/data/biomart)<br>Trimmomatic (v0.2)<br>CLC assembly cell (https://www.qiagenbioinformatics.com/products/clc-assembly-cell/)<br>Jellyfish (v2.2.6)<br>NLR-Annotator (https://github.com/steuernb/NLR-Annotator)<br>samtools (v1.9)<br>SnapGene (v5.0.7)

PROVEAN (v1.1)
Geneious Prime (v2022.2.2)
iTOL (https://itol.embl.de/)

For manuscripts utilizing custom algorithms or software that are central to the research but not yet described in published literature, software must be made available to editors and reviewers. We strongly encourage code deposition in a community repository (e.g. GitHub). See the Nature Portfolio guidelines for submitting code & software for further information.

Data

Policy information about availability of data

All manuscripts must include a data availability statement. This statement should provide the following information, where applicable:

- Accession codes, unique identifiers, or web links for publicly available datasets
- A description of any restrictions on data availability
- For clinical datasets or third party data, please ensure that the statement adheres to our policy

The RenSeq 150-bp paired-end Illumina sequences (raw data) for the 300 Watkins and 21 non-Watkins accessions (including Anahuac) and the cDNA RenSeq data of 6 Watkins accessions are available from NCBI study number PRJNA760793. The k-mer matrix and the CLC assemblies of the 300 Watkins and 21 non-Watkins accessions are available from Zenodo under the DOIs: 10.5281/zenodo.5557564, 10.5281/zenodo.5557685, 10.5281/zenodo.5557721, 10.5281/zenodo.5557827, 10.5281/zenodo.5557838 and 10.5281/zenodo.5655720. The genomic sequences of Rwt3 and Rwt4 are available from the wheat Ensembl databases as TraesCS1D02G029900 from https://plants.ensembl.org/Triticum_aestivum/ and TraesJAG1D03G00423590 from https://plants.ensembl.org/Triticum_aestivum_jagger/, respectively.

NLR and Kinase domains were predicted using NCBI (https://www.ncbi.nlm.nih.gov/Structure/cdd/wrpsb.cgi) and Pfam (http://pfam.xfam.org) databases.

Cadenza TILLING lines for Rwt4 candidate genes were identified using the Plant Ensembl database (http://plants.ensembl.org/Triticum_aestivum/)

Field-specific reporting

Please select the one below that is the best fit for your research. If you are not sure, read the appropriate sections before making your selection.

☒ Life sciences ☐ Behavioural & social sciences ☐ Ecological, evolutionary & environmental sciences

For a reference copy of the document with all sections, see nature.com/documents/nr-reporting-summary-flat.pdf

Life sciences study design

All studies must disclose on these points even when the disclosure is negative.

Sample size	No specific sample size experiment was done. A core set of 300 genetically diverse wheat landraces was chosen from the Watkins collection and the number was decided based on the available funding for sequencing.
Data exclusions	No deliberate data exclusion was done.
Replication	In the case of Watkins diversity panel, Br48+Pwt3 and Br48+Pwt4 phenotypes were scored for three replicates per genotype (Supplementary Table 3). In the case of Aegilops tauschii diversity panel, Br48+Pwt3 and Br48+Pwt4 phenotypes were scored for five replicates per genotype while Br48 phenotype was scored for three replicates per genotype (Supplementary Table 5).
Randomization	No deliberate randomization was imposed during the phenotype experiments. The phenotyping was done in a controlled environment where all of the exact same methods were used and scored by experienced researchers. The phenotypes for independent plants of the same genotype were generally consistent (see Supplementary Tables 3 and 5) and resulted in clear GWAS peaks (i) (in the case of Watkins diversity panel) around the loci that had been previously mapped by biparental genetics for Rwt3 and Rwt4, (ii) (in the case of Rwt4) around the same locus using two completely different diversity panels for wheat and Aegilops tauschii, and/or (iii) for which we confirmed the function of candidate genes, thus validating our methods and conclusions.
Blinding	The person doing the phenotyping did not have access to the genotype data. So in retrospect, the data collection was blinded.

Reporting for specific materials, systems and methods

We require information from authors about some types of materials, experimental systems and methods used in many studies. Here, indicate whether each material, system or method listed is relevant to your study. If you are not sure if a list item applies to your research, read the appropriate section before selecting a response.

Materials & experimental systems

n/a	Involved in the study
☒	☐ Antibodies
☒	☐ Eukaryotic cell lines
☒	☐ Palaeontology and archaeology
☒	☐ Animals and other organisms
☒	☐ Human research participants
☒	☐ Clinical data
☒	☐ Dual use research of concern

Methods

n/a	Involved in the study
☒	☐ ChIP-seq
☒	☐ Flow cytometry
☒	☐ MRI-based neuroimaging
