# Supplementary material for: A wheat kinase and immune receptor form host-specificity barriers against the blast fungus
Source: Nat Plants. 2023 Feb 16;9(3):385–92. doi: 10.1038/s41477-023-01357-5 (PMC10027608; doi:10.1038/s41477-023-01357-5)
Supplement: Supplementary file 5 — Rwt3 (exon1) alleles multiple sequence alignment. [file 41477_2023_1357_MOESM5_ESM.pdf]

F2: Multiple sequence alignment of the four groups of allelic variants of Rwt3 (first exon) identified in the *Ae. tauschii* and Watkins wheat panels.

```
I/Rwt3(exon1)      ATGGCGGCGGCTCTTGGTGCGGCGCAGTTGCTCCTCGGCAAGGTGCTCACGAAGCTCTCG    60
III(exon1)         ------------------------------------------------------------    0
II(exon1)          ------------------------------------------------------------    0
IV(exon1)          ------------------------------------------------------------    0


I/Rwt3(exon1)      GATGAGCGTATGGCCTCGTATGTGGCTAGCTCCGAGCTTGGCCTCGACTCCCAGAAGATC    120
III(exon1)         ------------------------------------------------------------    0
II(exon1)          ----------------------------GCTCTGAGCTTGGCCTCAACTCCCAACAGATC    32
IV(exon1)          ------------------------------------------------------------    0


I/Rwt3(exon1)      AAAGATGACATTATGTACACGGTTGGCCTGCTGCAGCAGGCCCAGGGGAGGGGCACCAGC    180
III(exon1)         ------------------------------------------------------------    0
II(exon1)          AAAGCTGGCCTCATGTACACGCAGGGCCTGCTGGTGGCGGCCGGCGGGAGGGCCATCGGC    92
IV(exon1)          ------------------------------------------------------------    0


I/Rwt3(exon1)      GACATTCCTGGCCTCACGGACTTGCTGGTGAAGCTAAGCCAGAAGGCCGATGAGGCTGAG    240
III(exon1)         ------------------------------------------------------------    0
II(exon1)          GACAGCCCTGGCCTGCAGGGGTTGTTGGCGGAGCTGAGCAACAAGGCTGACGAGGCGGAG    152
IV(exon1)          ------------------------------------------------------------    0


I/Rwt3(exon1)      AAAGTGCTGAACGAGCTCCGGTACTTCATGATCCGGGACCAGATTAACGGCACCCAGCTA    300
III(exon1)         ------------------------------------------------------------    0
II(exon1)          ATTGCCCTGAATGAGCTCCACTACTTCATGATCCAGGACCAGCTCGACGGTACCATCAAC    212
IV(exon1)          ------------------------------------------------------------    0


I/Rwt3(exon1)      GTCAAGCCAGATCTGGGCGCTTGCCTCAAGACCAAGAA------------AGGTCATGCT    348
III(exon1)         ---------------------------------------------------------GCT    3
II(exon1)          GCTGTGCCAGATCTGGATGTGGGCATCCGGGGACATGCTGATCATGGCGGCCATGCAGCT    272
IV(exon1)          ---------------------------------------------------------GCT    3
                                                                            ***

I/Rwt3(exon1)      CGTCACACTATTGGTAACTGTCTGCCATTCATTTTTCGTCCATGTACCAGTAGCCAACAA    408
III(exon1)         CGTCACGCTATTGGTAACTGGCTTCCACTCTTTTCCTGCTCGTGCGTCAAGAACCCACAC    63
II(exon1)          CGTCACGCTATTGGTAACTGGCTTCCGCTCTTTTCCTGCTCGTGCGTCAAGAACCCACAC    332
IV(exon1)          CGTCACGCTATTGGTAACTGGCTTCCACTCTTTTCCTGCTCGTGCGTCAAGAACCCACAC    63
                   ****** ************* ** **  ** ***   *  * **   **  * ** ***

I/Rwt3(exon1)      GATGGAACCATGTCTGATGGCCATGACCATGTTGATGAGTTGCCATTTGATAGTGTGTCC    468
III(exon1)         AAGGAAACCAAGTCTGAGATTGGCGGCGATGCTGATGTGTTGCCATTTGATAGAGTGGCT    123
II(exon1)          AAGGAAACCAAGTCTGAGATTGGCGGCGATGCTGATGTGTTGCCATTTGATAGAGTGGCT    392
IV(exon1)          AAGGAAACCAAGTCTGAGATTGGCGGCGATGCTGATGTGTTGCCATTTGATAGAGTGGCT    123
                    * * ***** ******       * * *** ***** *************** *** *

I/Rwt3(exon1)      ATATCCATCAAAATCAAGTCGGTGTTAGAGGAGACACACTCCATATGT---GTTCATGTC    525
III(exon1)         ATGTCCATGAAAATTAAGTCACTGACAGAGAGCATAAATTTTATGTTGTGCACTGCTGTC    183
II(exon1)          ATGTCCATGAAAATTAAGTCACTGACAGAGAGCATAAAATTTAT---GTGCACTGCTGTC    449
IV(exon1)          ATGTCCATGAAAATTAAGTCACTGACAGAGAGCATAAATTTTAT---GTGCACTGCTGTC    180
                   ** ***** ***** *****  **  ****   * * * *  **         *  ****

I/Rwt3(exon1)      TCCGACTTGCTCAAGTTGATTCCAAACCACGGCAGCAGCTCTACGACAGCCACAACTGTC    585
III(exon1)         TCCGACTTGCTCAGAATAGTTGCAGC------------AACACATAGCAGCACCACTGTC    231
II(exon1)          TCCGACTTGCTCAGAATAGTTGCAGC------------AACACATAGCAGCACCACTGTC    497
IV(exon1)          TCCGACTTGCTCAGAATAGTTGCAGC------------AACACATAGCAGCACCACTGTC    228
                   *************   *  ** **                *    *    *** ******

I/Rwt3(exon1)      ACCCGCATACGGCCTACTAGAGGATCAATGGTTGCACAGGATACAATGTATGGTAGGCGG    645
III(exon1)         GCCCTGAAACGGCCTCTTGTAGGATCAACGCTTTCACAAGATAGACTGTATGGTAGAGAG    291
II(exon1)          GCCCTGAAACGGCCTCTTGTAGGATCAACGATTTCACAAGATAGACTGTATGGTAGAGAG    557
IV(exon1)          GCCCTGAAACGGCCTCTTGTAGGATCAACGATTTCACAAGATAGACTGTATGGTAGAGAG    288
                    ***  * *******  *  ******** * ** **** **** * **********   *

I/Rwt3(exon1)      GACATCTTTGAGAA-AACTATACATGCCATCACCAGTTGCGGCGAAATCCTTTCTGTTCT    704
III(exon1)         GACATTTTTGAGAA-AACAATAGATTGTATCATCAGTTGCAGTGAAACCCTTCATGTTAT    350
II(exon1)          GACATTTTTGAGAAAAAACATAGATTGTATCATCAGTTGCAGTGAAACCCTTTATGTTAT    617
IV(exon1)          GACATTTTTGAGAAAAAACATAGATTGTATCATCAGTTGCAGTGAAACCCTTTATGTTAT    348
                   ***** ******** **  *** **   **** ******* * **** ****  **** *
```

```
I/Rwt3(exon1)       CCCTGTAGTTGGGCCAGGGGGCATTGGAAAAACAACTTTAATCCAACACCTGTATAATGA    764
III(exon1)          TCCCATACTTGGACCAGGCGGTATTGGAAAATCAACATTCACCCAACACCTGTATAATGA    410
II(exon1)           TCCCATACTTGGACCAGGCGGTATTGGAAAATCAACATTCACCCAACACCTGTATAATGA    677
IV(exon1)           TCCCATACTTGGACCAGGCGGTATTGGAAAATCAACATTCACCCAACACCTGTATAATGA    408
                     **  ** **** ***** ** ********* **** ** * ******************

I/Rwt3(exon1)       TAAAAGGATTGAAGAACACTTTGATGTCAGAATATGGGTATGTGTATCAATTGATTTTGC    824
III(exon1)          TACAAGGACTCAACAACACTTTAGTGTCAGGGTCTGGATATCTGTGTCCACTGATTTTGA    470
II(exon1)           TACAAGGACTCAACAACGCTTTAGTGTCAGGGTCTGGATATCTGTGTCCACTGATTTTGA    737
IV(exon1)           TACAAGGACTCAACAACGCTTTAGTGTCAGGGTCTGGATATCTGTGTCCACTGATTTTGA    468
                    ** ***** * ** *** ****  ******  * *** *** *** ** * ********

I/Rwt3(exon1)       TGTGCTTAAGCTCACCC----GGGAGATCTTTAGATGCATAATTGCAACTGAAGAAGAAG    880
III(exon1)          TGTGCTTAAGCTAATTAACCCAGCAAATCCTTAGCTGCATACCTGCAACTGAAGAAGAAG    530
II(exon1)           TGTGCTTAAGCTAATTAACCCAGCAAATCCTTAGCTGCATACCTGCAACTGAAGAAGAAG    797
IV(exon1)           TGTGCTTAAGCTAATTAACCCAGCAAATCCTTAGCTGCATACCTGCAACTGAAGAAGAAG    528
                    ************ *        * * *** **** ******  *****************

I/Rwt3(exon1)       GAAGAAATAGTGCTCCC----AGTGAAACAGCCAGTATAGAACAGCTTCAGGAGTCTATT    936
III(exon1)          ATAGCAACACTGCACATGTAAGATGTAACAAACAGCTTAAGCAACATTCAAGAATACATT    590
II(exon1)           ATAGCAACACTGCACATGTAAGATGTAACAAACAGCTTAAGCAACATTCAAGAATACATT    857
IV(exon1)           ATAGCAACACTGCACATGTAAGATGTAACAAACAGCTTAAGCAACATTCAAGAATACATT    588
                      ** ** * *** *        ** ****  ***  **    *  **** ** *  ***

I/Rwt3(exon1)       GCACAGAGGCTCCAGTCCAAAAGGTTTTTAATTGTTCTGGATGATGTGTGGCAACTCAAT    996
III(exon1)          GCACTGAGACTGAGATCTAAAAGGGTTTTAGTAGTCTTGGATGATGTATGGAAATGTGAT    650
II(exon1)           GCACTGAGACAGAGATCTAAAAGGGTTTTAGTAGTCTTGGATGATGTATGGAAATGTGAT    917
IV(exon1)           GCACTGAGACTGAGATCTAAAAGGGTTTTAGTAGTCTTGGATGATGTATGGAAATGTGAT    648
                    **** *** *     ** ****** ***** * **  ********** *** **    **

I/Rwt3(exon1)       AGTGAAGATGAGTGGCATACCCTATTAGCTCCATTCAAAAAGGGAGGAGCCAAGGGCAAC    1056
III(exon1)          AGTGTGGAAGACTGGAAAACCCTATTAGCTCCGTTCACAATGGGGGAAGCCAAAGGCAGC    710
II(exon1)           AGTGTGGAAGACTGGAAAACCCTATTAGCTCCGTTCACAATGGGGGAAGCCAAAGGCAGC    977
IV(exon1)           AGTGTGGAAGACTGGAAAACCCTATTAGCTCCGTTCACAATGGGGGAAGACAAAGGCAGC    708
                    ****  ** ** *** * ************** **** ** *** * ** *** **** *

I/Rwt3(exon1)       ATGGTACTTGTCACAACCCGGTTCCCATCTATAGCAGGCAGGGTGAAGACAGTTGATCCA    1116
III(exon1)          ATGGTACTTGTCACAACTCGATTTCCAAAACCAGCAGAAATGATGAAAACAGTTGATCAA    770
II(exon1)           ATGGTACTTGTCACAACTCGATTTCCAAAACCAGCAGAAATGATGAAAACAGTTGATCAA    1037
IV(exon1)           ATGGTACTTGTCACAACTCGATTTCCAAAACCAGCAGAAATGATGAAAACAGTTGATCAA    768
                    ***************** ** ** ***     *****  * * **** ********** *

I/Rwt3(exon1)       ------GTCGAGCTGCAAGGTTTAGACACTGATGATTTCTTCGCATTCTTTGAAGAGTGT    1170
III(exon1)          CTGCCACTAGAACTGCAAGGTTTGGAGCCTAATGACTTCTTCGCATTCTTTGAAGCATGC    830
II(exon1)           CTGCCACTAGAACTGCAAGGTTTGGAGCCTAATGACTTCTTCGCATTCTTTGAAGCATGC    1097
IV(exon1)           CTGCCACTAGAACTGCAAGGTTTGGAGCCTAATGACTTCTTCGCATTCTTTGAAGCATGC    828
                           * ** *********** **  ** **** ******************* **

I/Rwt3(exon1)       ATATTTGGTGGACTCGATAAGCCTATGCATTACAGAGATGAGTTAACTGATATTGCAAGA    1230
III(exon1)          ATATTTGGTGAACTCGATAGACCTGAAGGTTACACAGACATTTTAAGTGACATTGCTAGA    890
II(exon1)           ATATTTGGTGAACTCGATAGACCTGAAGGTTACACAGACATTTTAAGTGACATTGCTAGA    1157
IV(exon1)           ATATTTGGTGAACTCGATAGACCTGAAGGTTACACAGACATTTTAAGTGACATTGCTAGA    888
                    ********** ********  ***     ***** ***    **** *** ***** ***

I/Rwt3(exon1)       GATATTGTAGATAAGTTAAAGGGTTCACCTTTAGCAGCCAAAACAGTCGGCCGCCTATTA    1290
III(exon1)          AAAGTTGCATATAAGTTAATGGGTAACCCTCTAGCAGCCAAAACAGCCAGTCGGCTATTG    950
II(exon1)           AAAGTTGCATATAAGTTAATGGGTAACCCTCTAGCAGCCAAAACAGCCAGTCGGCTATTG    1217
IV(exon1)           AAAGTTGCATATAAGTTAATGGGTAACCCTCTAGCAGCCAAAACAGCCAGTCGGCTATTG    948
                     *  *** * ********* ****   *** *************** * * ** *****

I/Rwt3(exon1)       AGGAAAGACCTATCCTGGGAACACTGGACAAGAGTTCTTGAAAGCATGGAATGGGAAAAT    1350
III(exon1)          AGGAAAGACATGTCTCAGGAACATTGGATGGGAGTTCTTGAAAACAAGGAATGGGAACAT    1010
II(exon1)           AGGAAAGACATGTCTCAGGAACATTGGATGGGAGTTCTTGAAAACAAGGAATGGGAACAT    1277
IV(exon1)           AGGAAAGACATGTCTCGGGAACATTGGATGGGAGTTCTTGAAAACAAGGAATGGGAACAT    1008
                    ********* * **   ****** ****   ************ ** ********** **

I/Rwt3(exon1)       AAGCAGAGCGATAATGATACTATGCCAGCTTTGTACCTTAGCTACGATTACCTCCCTTTC    1410
III(exon1)          AGGCAAAACGATGACGATATTATGTCATCACTGAGCACTAGCTACGATTACCTCCCTTTT    1070
II(exon1)           AGGCAAAACGATGACGATATTATGTCATCACTGAGCACTAGCTACGATTACCTCCCTTTT    1337
IV(exon1)           ACGCAAAACGATGACGATATTATGTCATCACTGAGCACTAGCTACGATTACCTCCCTTTT    1068
                    * *** * **** * **** **** ** *  **  *  *********************

I/Rwt3(exon1)       CTCCTGCAAAAATGTTTTTCCTATCTTTCTCTTTTTCCTGAAGATCATAGGTTTAGTCAT    1470
III(exon1)          CTCCAGCAAAAATGCTTTTCCTACTGTGCCCTTTTCCCTAAAGGTCATAGGTTTAGTCAT    1130
II(exon1)           CTCCAGCAAAAATGCTTTTCGTACTGTGCCCTTTTCCCTAAAGGTCATAGGTTTAGTCAT    1397
IV(exon1)           CTCCAGCAAAAATGCTTTTCGTACTGTGCCCTTTTCCCTAAAGGTCATAGGTTTAGTCAT    1128
                    **** ********* ***** **   * * ***** *** *** ****************
```

```
I/Rwt3(exon1)      TCAGAAATTAATCGCTTTTGGATTGCAGTAG----GCATCATAGACTCTAATCATCCAGG   1526
III(exon1)         TTAGAAATGAATCGTTTTTTGACTGCGGTAGCTAGGCATCATAGATTTTTCTCACCAAGG   1190
II(exon1)          TTAGAAATGAATCT-TTTTTGACTGCGGTAGCTAGGCATCATAGATTTTTCTCACTAAGG   1456
IV(exon1)          TTAGAAATGAATCGTTTTTTGACTGCGGTAGCTAGGCATCATAGATTTTTCTCACCAAGG   1188
                   * ****** ****  **** ** *** ****    ********** * *  ***   ***

I/Rwt3(exon1)      AAATAACAATTGCTTGGAAGAACTAGTGGACCGTGGTTTTCTTAGGAGGGTACATGGTTT   1586
III(exon1)         CGCTAAAACCTATTTGGAAGACCTAGTGGACAATGGTTTTCTTGTTAAGGCAGATGATTT   1250
II(exon1)          CGCTAAAACCTATTTGGAAGACCTAGTGGACAATGGTTTTCTTGTTAAGGCAGATGATTT   1516
IV(exon1)          CGCTAAAACCTATTTGGAAGACCTAGTGGACAATGGTTTTCTTGTTAAGGCAGATGATTT   1248
                      *** *  *  ******** *********  **********   * ** * *** ***

I/Rwt3(exon1)      GTTTGGTGAAGAATACTATGTAATCCATGATTTACTGCATGAACTATCTCGAAGTGTTTC   1646
III(exon1)         GATTGGTC---AAAACTATATAATGCCTAATTTATGGCATGACCTATCTCGAAATGTTTC   1307
II(exon1)          GATTGGTC---AAAACTATATAATGCCTAATTTATTGCATGACCTATCTCGAAATGTTTC   1573
IV(exon1)          GATTGGTC---AAAACTATATAATGCCTAATTTATGGCATGACCTATCTCGAAATGTTTC   1305
                   * *****    ** ***** **** * * *****  ****** ********** ******

I/Rwt3(exon1)      AACACAAGAATGCCTCAATATAAGTGTTTTAGATTTTAAAGCTGACACAGTCCCACCATC   1706
III(exon1)         AGCACATGAATGCCTAAATATAAGTGGATTAGATTTTAAAGCTGACACAGTCCCACAATC   1367
II(exon1)          AGCACATGAATGCCTAAATATAAGTGGATTAGATTTTAAAGCTGACACAGTCCCACAATC   1633
IV(exon1)          AGCACATGAATGCCTAAATATAAGTGGATTAGATTTTAAAGCTGACACAGTCCCACAATC   1365
                   * **** ******** **********  **************************** ***

I/Rwt3(exon1)      TATTCAACACTTATCTATCACCCTAGAAAATAAATATGATGGAATTTTTTTGGAAGAAAT   1766
III(exon1)         TATTCGACACCTGTCTATCACTTTGAAAGATACACATGATGAAAATTTTCTTCAAGAAGT   1427
II(exon1)          TATTCGACACCTGTCTATCACTTTGAAAGATACACATGATGAAAATTTTCTTCAAGAAGT   1693
IV(exon1)          TATTCGACACCTGTCTATCACTTTGAAAGATACACATGATGAAAATTTTCTTCAAGAAGT   1425
                   ***** **** * ********  *  ** *** * ****** ** **** *  ***** *

I/Rwt3(exon1)      AAGTAAACTGAAAATAAAGATAGACATTGCAAATTTACGGACTTTAATGATTTTTAGTGC   1826
III(exon1)         GAGTAAACTAAAAACTAGGATAGACATTGCAAATTTACGAACTTTGATGATTTTTCATGA   1487
II(exon1)          GAGTAAACTAAAAACTAGGATAGACATTGCAAATTTACGAACTTTGATGATTTTTCATGA   1753
IV(exon1)          GAGTAAACTAAAAACTAGGATAGACATTGCAAATTTACGAACTTTGATGATTTTTCATGA   1485
                    ******** ****  * ********************* ***** *********  **

I/Rwt3(exon1)      ATATGAAGAAAGAATTGCTGGTATTTTAAAAGATACGTTTGAGGAAGTAGATAGTCTGCA   1886
III(exon1)         ATATGAAGGAAAAATTGCAGAGATTTTAAAAGATACGTTTAAGGAACTAAATAATCTGAA   1547
II(exon1)          ATATGAAGGAAAAATTGCAGAGATTTTAAAAGATACGTTTAAGGAACTAAATAATCTGAA   1813
IV(exon1)          ATATGAAGGAAAAATTGCAGAGATTTTAAAAGATACGTTTAAGGAACTAAATAATCTGAA   1545
                   ******** ** ****** *  ****************** ***** ** *** **** *

I/Rwt3(exon1)      TGTCCTATTTATAGTTGTGAAATCCCTGGATGATTTGCCAAAAGGCTTTTCAAAACTTAT   1946
III(exon1)         TGTACTATTTATAGTTGTGAAATCCTTAGATGATTTACCACAA----------AACTTAT   1597
II(exon1)          TGTACTATTTATAGTTGTGAAATCCTTAGATGATTTACCACAA----------AACTTAT   1863
IV(exon1)          TGTACTATTTATAGTTGTGAAATCCTTAGATGATTTACCACAA----------AACTTAT   1595
                   *** ********************* * ******** *** **          *******

I/Rwt3(exon1)      CCACCTCCAGTACCTCAAACTTGGATCACCTATTGGCATAGAAATGGCATTACCTAGCAC   2006
III(exon1)         CCACCTCCAGTACCTCAAAGTTGGATCACCTTACGGCGTTGAAATAACTTTACCTAGTAC   1657
II(exon1)          CCACCTCCAGTACCTCAAAGTTGGATCACCTTACGGCGTTGAAATAACTTTACCTAGTAC   1923
IV(exon1)          CCACCTCCAGTACCTCAAAGTTGGATCACCTTACGGCGTTGAAATAACTTTACCTAGTAC   1655
                   ******************* ***********   *** * *****  * ******** **

I/Rwt3(exon1)      ACTGGCCAGATTTTATCACTTGAAATTCTTAGACCTAAAAGATTGGCATGGTAGTTCTAA   2066
III(exon1)         ACTATCCAGATTTTATCACTTGAAATTCTTAGACCTGGAAGATTGGCATGGTAGTTCTAA   1717
II(exon1)          ACTATCCAGATTTTATCACTTGAAATTCTTAGACCTGGAAGATTGGCATGGTAGTTCTAA   1983
IV(exon1)          ACTATCCAGATTTTATCACTTGAAATTCTTAGACCTGGAAGATTGGCATGGTAGTTCTAA   1715
                   ***  *******************************  **********************

I/Rwt3(exon1)      TGTTCCTAAAGACATTAGTCACCTTGTGAATTTGCAAGACTTCATTGCTAAAAAAGAACT   2126
III(exon1)         GTTGCCTAAAGACATTAACCGCCTAGTGAATTTGCAAGACTTCCTTGCTAAAAAAGAACT   1777
II(exon1)          GTTGCCTAAAGACATTAACCGCCTAGTGAATTTGCAAGACTTCCTTGCTAAAAAAGAACT   2043
IV(exon1)          GTTGCCTAAAGACATTAACCGCCTAGTGAATTTGCAAGACTTCCTTGCTAAAAAAGAACT   1775
                     * *************  * *** ****************** ****************

I/Rwt3(exon1)      CCACTCCAGTGTTCTTGAGGTTGGAAAGATGAAGTACCTACGGGAACTAAAACAATTCTG   2186
III(exon1)         CCACTCCAGTGTTCCTGAGGTTGGAAAGATGAAGTGTCTACAGGAATTAAAAGAATTCTG   1837
II(exon1)          CCACTCCAGTGTTCCTGAGGTTGGAAAGATGAAGTGTCTACAGGAATTAAAAGAATTCTG   2103
IV(exon1)          CCACTCCAGTGTTCCTGAGGTTGGAAAGATGAAGTGTCTACAGGAATTAAAAGAATTCTG   1835
                   ************** ********************  **** **** ***** *******

I/Rwt3(exon1)      TGTTAAGAAAGAGAGTGTTGGGTTTGAATTAAGAGAGCTAGGGGAACTGACAGAGCTTGG   2246
III(exon1)         TGTTAAGAAAGAGAGAGTTGGATTCGAATTAAGAGAGCTGGGGGAAATGACAGAGCTTGG   1897
II(exon1)          TGTTAAGAAAGAGAGAGTTGGATTCGAATTAAGAGAGCTGGGGGAAATGACAGAGCTTGG   2163
IV(exon1)          TGTTAAGAAAGAGAGAGTTGGATTCGAATTAAGAGAGCTGGGGGAAATGACAGAGCTTGG   1895
```

```
                    *************** ***** ** ************** ****** *************

I/Rwt3(exon1)       GGGAGAACTCAGAATATGTAACCTTGAAAACGTGGCAACCAAGGAAGAAGCTAGTGAGGC   2306
III(exon1)          AGGAGAACTCAGAATATGCAACCTCGAAAACGTGGCAACCAAGGAAGAAGCTAGTGAAGC   1957
II(exon1)           AGGAGAACTCAGAATATGCAACCTCGAAAACGTGGCAACCAAGGAAGAAGCTAGTGAAGC   2223
IV(exon1)           AGGAGAACTCAGAATATGCAACCTCGAAAACGTGGCAACCAAGGAAGAAGCTAGTGAAGC   1955
                     ***************** ***** ******************************** **

I/Rwt3(exon1)       CAAACTGATGTCGAAAAGGAATCTGAAGAAGCTGACATTGGTTTGGGGCAGAAAACAATC   2366
III(exon1)          CAAACTGCTGTCAAAAAAGAATCTGAAAAAGTTGACATTGGTTTGGGGCAGAAAACAACC   2017
II(exon1)           CAAACTGCTGTCAAAAAAGAATCTGAAAAAGTTGACATTGGTTTGGGGCAGAAAACAACC   2283
IV(exon1)           CAAACTGCTGTCAAAAAAGAATCTGAAAAAGTTGACATTGGTTTGGGGCAGAAAACAACC   2015
                    ******* **** **** ********* *** ************************** *

I/Rwt3(exon1)       GACTATAGATGGTGATGTTCTTGATGCTCTTCAACCGCATCCTGATCTTAGAGAGCTTCG   2426
III(exon1)          AACTATAGATGCTGATGTTCTTCATGGTTTTCGACCACATCCTTATCTTAGAGAACTGTG   2077
II(exon1)           AACTATAGATGCTGATGTTCTTCGTGGTTTTCGACCACATCCTTATCTTAGAGAACTGTG   2343
IV(exon1)           AACTATAGATGCTGATGTTCTTCGTGGTTTTCGACCACATCCTTATCTTAGAGAACTGTG   2075
                     ********** **********  ** * *** *** ****** ********** **  *

I/Rwt3(exon1)       AATTGCAAATCATGGTGGTGCTGTTGGTCCCAGTTGGTTGTGTGTTGACATGTTGCTGAA   2486
III(exon1)          CATTGCAAATCATGATGGTGCGGCTGGTCCCAGTTGGCTGTGTGTTGACATTTTGGTGAA   2137
II(exon1)           CATTGCAAATCATGATGGTGCGGCTGGTCCCAGTTGGCTGTGTGTTGACATTTTGGTGAA   2403
IV(exon1)           CATTGCAAATCATGATGGTGCGGCTGGTCCCAGTTGGCTGTGTGTTGACATTTTGGTGAA   2135
                     ************* ****** * ************* ************* *** ****

I/Rwt3(exon1)       ACAGTTAGGTGTTCTCCATCTCGAAGGTTTGTCTTGGGACACTCTTCCACCTTTTGGGCA   2546
III(exon1)          ACATTTAGAGGCTATCCATCTCGAAGGGCTGTCATGGGATGCTCTTCCACCTTTCAGGCA   2197
II(exon1)           ACATTTAGAGGCTATCCATCTCGAAGGGCTGTCATGGGATGCTCTTCCACCTTTCAGGCA   2463
IV(exon1)           ACATTTAGAGGCTATCCATCTCGAAGGGCTGTCATGGGATGCTCTTCCACCTTTCAGGCA   2195
                    *** ****  * * *************  **** *****  *************  ****

I/Rwt3(exon1)       GCTACCACGCCTCACTAAACTCATTTTGATGAGGATTTCTGGAGTGCATCAG--------   2598
III(exon1)          GATACCACACCTCAGAAAACTCATTTTGACGAGGATTTCTGGAGTGCATCAGTTAGGAAT   2257
II(exon1)           GATACTACACCTCAGAAAACTCATTTTGACGAGGATTTCTGGAGTGCATCAGTTAGGAAT   2523
IV(exon1)           GATACCACACCTCAGAAAACTCATTTTGACGAGGATTTCTGGAGTGCATCAGTTAGGAAT   2255
                    * *** ** *****  ************* **********************

I/Rwt3(exon1)       ------------------------------------------------------------   2598
III(exon1)          CCAGGATATGCTGCGCCTGGAGTCATTAATGATATGCAGATGTGGAAACTTCTTCTCAGG   2317
II(exon1)           CCAGGATATGCTGCGCCTGGAGTCATTAATGATATGCAGATGTGGAAACTTCTTCTCAGG   2583
IV(exon1)           CCAGGATATGCTGCGCCTGGAGTCATTAATGATATGCAGATGTGGAAACTTCTTCTCAGG   2315


I/Rwt3(exon1)       ------------------------------------------------------------   2598
III(exon1)          GTGTTCCACGGAAGAAGCAGGAGGAACCCATACCATGAAGCCTTTCCCTGCTTACCTCAA   2377
II(exon1)           GTGTTCCACGGAAGAAGCAGGAGGAACCCATACCATGAAGCCTTTCCCTGCTTACCTCAA   2643
IV(exon1)           GTGTTCCACGGAAGAAGCAGGAGGAACCCATACCATGAAGCCTTTCCCTGCTTACCTCAA   2375


I/Rwt3(exon1)       -------------------------------------TTGGCTCTGCTCTCAAACCTCAC   2621
III(exon1)          GAAGCTTGGCATTTCATGTGAGTCAAGCATGCAGTCAATGGCTCTGCTCTCAAACCTTAC   2437
II(exon1)           GAAGCTTGGCATTTCATGTGAGTCAAGCATACAGTCAATGGCTCTGCTCTCAAACCTTAC   2703
IV(exon1)           GAAGCTTGGCATTTCATGTGAGTCAAGCATACAGTCAATGGCTCTGCTCTCAAACCTTAC   2435
                                                         ******************* **

I/Rwt3(exon1)       GTCTCTCACCCATCTGACACTGCAAGATTGTGTCAAGTTAACAGTGGATGGATTCAATCC   2681
III(exon1)          ATCCCTCACCCATCTGACACTACAAGACTGTGTTAAGTTAACAGTGGATGGGTTCAATCC   2497
II(exon1)           ATCCCTCACCCATCTGACACTACAAGACTGTGTTAAGTTAACAGTGGATGGGTTCAATCC   2763
IV(exon1)           ATCCCTCACCCATCTGACACTACAAGACTGTGTTAAGTTAACAGTGGATGGGTTCAATCC   2495
                     ** ***************** ***** ***** ***************** ********

I/Rwt3(exon1)       TCTTACCACAGTCAACCTTAAGGTATTGGTGGTATTTAACTGCCGATGGGACAGAAGTTG   2741
III(exon1)          TCTCATCACAGTCAACCTCAAGGCATTGGTGGTATTTAACTGCAGATGGGACAGAAGTTG   2557
II(exon1)           TCTCATCACAGTCAACCTCAAGGCATTGCTGGTATTTAACTGCAGATGGGACAGAAGTTG   2823
IV(exon1)           TCTCATCACAGTCAACCTCAAGGCATTGGTGGTATTTAACTGCAGATGGGACAGAAGTTG   2555
                    *** * ************ **** **** ************** ****************

I/Rwt3(exon1)       TCCCGAATCTGTAGCAGCGGATCTTCTCACAAAGGTGGCAAGTAGCAGAGTAATGCCTGC   2801
III(exon1)          TCCTGAATCAATAGCAGCAGCTCTGCTCTCAGATGTGGCAAGTAGCAGAATAATGCCTGC   2617
II(exon1)           TCCTGAATCAATAGCAGCAGCTCTGCTCTCAGATGTGGCAAGTAGCAGAATAATGCCTGC   2883
IV(exon1)           TCCTGAATCAATAGCAGCAGCTCTGCTCTCAGATGTGGCAAGTAGCAGAATAATGCCTGC   2615
                    *** *****  ******* * *** *** ** * *************** **********

I/Rwt3(exon1)       AGGTTCCTTCCGATTAGAACAGCTCAAGGTGGACAGCATCTCGGCAGTGCTTGTCACTCC   2861
III(exon1)          AGGTTCCTTCCAGTTGGAACAGCTCAAGGTGGATAGCATCCCTGCAGTGCTTGTCACTCG   2677
II(exon1)           AGGTTCCTTCCAGTTGGAACAGCTCAAGGTGGATAGCATCCCTGCAGTGCTTGTCACTCG   2943
```

```
IV(exon1)           AGGTTCCTTCCAGTTGGAACAGCTCAAGGTGGATAGCATCCCTGCAGTGCTTGTCACTCG    2675
                    ***********  ** ***************** ****** * ****************

I/Rwt3(exon1)       CATCTGCAACCTTGTCGCCCATACCCTCCAGACTCTGATATTCTGCCATGATCACCGGAT    2921
III(exon1)          CATCTGCAACCTAATTGCCACTACCCTTCACACTCTGATATTCTGCCATGATCACCGGGC    2737
II(exon1)           CATCTGCAACCTAATTGCCACTACCCTTCACACTCTGATATTCTGCCATGATCACCGGGC    3003
IV(exon1)           CATCTGCAACCTAATTGCCACTACCCTTCACACTCTGATATTCTGCCATGATCACCGGGC    2735
                    ************  * ***  ****** ** ***************************

I/Rwt3(exon1)       CAAGAGTTTCACAGAAGAGCAAGAGAAGGCGCTTCTGCTCCTCACATCACTCCGACACCT    2981
III(exon1)          CAAAAGCTTCACAGAAGAGCAAGAGAAGGCGCTTCAGTTCCTCACTTCCCTCCGACACCT    2797
II(exon1)           CAAAAGCTTCACAGAAGAGCAAGAGAAGGCGCTTCAGTTCCTCACTTCCCTCCGACACCT    3063
IV(exon1)           CAAAAGCTTCACAGAAGAGCAAGAGAAGGCGCTTCAGTTCCTCACTTCCCTCCGACACCT    2795
                    *** ** **************************** * ******* ** ***********

I/Rwt3(exon1)       CACATTTGATGGTTGTGGGGCTCTGCAGTCCCTCCCGAGAGGGTTGAATCGCCTTTCTTC    3041
III(exon1)          CAGGTTTGATGGATGTGGGGCTCTGCAGTCCCTCCCGCGAGGGTTGAGTCACCTTTCTTC    2857
II(exon1)           CAGGTTTGATGGATGTGGGGCTCTGCAGTCCCTCCCGCGAGGGTTGAGTCACCTTTCTTC    3123
IV(exon1)           CAGGTTTGATGGATGTGGGGCTCTGCAGTCCCTCCCGCGAGGGTTGAGTCACCTTTCTTC    2855
                    **  ******** ************************ ********* ** *********

I/Rwt3(exon1)       ACTCAAAGACTTAGAGGTCCTCTGGTGCCCTGAAATGGGATCCATACCCAAGGAGGGGTT    3101
III(exon1)          TCTCGAGGGCTTAGAGGTCCTTTGGTGTCCTGAACTTCAATCCATACCCAAGGAGGGCTT    2917
II(exon1)           TCTCGAGGGCTTAGAGGTCCTTTGGTGTCCTGAACTTCAATCCATACCCAAGGAGGGCTT    3183
IV(exon1)           TCTCGAGGGCTTAGAGGTCCTTTGGTGTCCTGAACTTCAATCCATACCCAAGGAGGGCTT    2915
                     *** * * ************ ***** ****** *   ****************** **

I/Rwt3(exon1)       CCCAGTTTCGCTCGAAATCCTACGCATAAGACCTTGCAGCCCCGAGGTTAGGGAGCAAAT    3161
III(exon1)          CTCCATTTCGCTGAGAAACCTACGCATAAGGCCTTGCAGCACCGAGGTTAAGGAGCAAAT    2977
II(exon1)           CTCCATTTCGCTGAGAAACCTACGCATAAGGCCTTGCAGCACCGAGGTTAAGGAGCAAAT    3243
IV(exon1)           CTCCATTTCGCTGAGAAACCTACGCATAAGGCCTTGCAGCACCGAGGTTAAGGAGCAAAT    2975
                    * *  *******   ** ************ ********* ********* *********

I/Rwt3(exon1)       TGAGAAACTCAGAAGAACAAGCCCAGGTTTATCTGTACGATACGAGTAA       3210
III(exon1)          TGAGAAACTCAGAAGAACAAGCCCAGGTTTATCTGTACAATACGAGTAA       3026
II(exon1)           TGAGAAACTCAGAAGAACAAGCCCAGGTTTATCTGTACAATACGAGTAA       3292
IV(exon1)           TGAGAAACTCAGAAGAACAAGCCCAGGTTTATCTGTACAATACGAGTAA       3024
                    ************************************** **********
```
