# Supplementary material for: A wheat kinase and immune receptor form host-specificity barriers against the blast fungus
Source: Nat Plants. 2023 Feb 16;9(3):385–92. doi: 10.1038/s41477-023-01357-5 (PMC10027608; doi:10.1038/s41477-023-01357-5)
Supplement: Supplementary file 6 — Rwt4 NLR alleles multiple sequence alignment. [file 41477_2023_1357_MOESM6_ESM.pdf]

F3: Multiple sequence alignment of the five groups of allelic variants of the *Rwt4* NLR candidate identified in the *Ae. tauschii* and Watkins wheat panels

```
IV              ATGGAGGAGATCGTGCTTGGACTGTCCAAGACGGTGGTGGAGGGGACGCTGGTCAAGGTG      60
V               ATGGAGGAGATCGTGCTTGGACTGTCCAAGACGGTGGTGGAGGGGACGCTGGTCAAGGTG      60
I/Rwt4NLR       ATGGAGGAGATCGTGCTTGGACTGTCCAAGACGGTGGTGGAGGGGACGCTGGTCAAGGTG      60
II              ATGGAGGAGATCGTGCTTGGACTGTCCAAGACGGTGGTGGAGGGGACGCTGGTCAAGGTG      60
III             ATGGAGGAGATCGTGCTTGGACTGTCCAAGACGGTGGTGGAGGGGACGCTGGTCAAGGTG      60
                ************************************************************

IV              AAGGCCGCGATTGACGAGGAGGCGAAGCTGAAGCTTGCCGTGCAGAGCGACCTGGTGTTC      120
V               AAGGCCGCGATTGACGAGGAGGCGAAGCTGAAGCTTGCCGTGCAGAGCGACCTGGTGTTC      120
I(Rwt4NLR)      AAGGCCGCGATTGACGAGGAGGCGAAGCTGAAGCTTGCCGTGCAGAGCGACCTGGTGTTC      120
II              AAGGCCGCGATTGACGAGGAGGCGAAGCTGAAGCTTGCCGTGCAGAGCGACCTGGTGTTC      120
III             AAGGCCGCGATTGACGAGGAGGCGAAGCTGAAGCTTGCCGTGCAGAGCGACCTGGTGTTC      120
                ************************************************************

IV              ATCACGGGGGAATTCGAGATGATGCAGTCGTTCCTCAACGTCGCCGACGCGGAGCGCATC      180
V               ATCACGGGGGAATTCGAGATGATGCAGTCGTTCCTCAACGTCGCCGACGCGGAGCGCATC      180
I(Rwt4NLR)      ATCACGGGGGAATTCGAGATGATGCAGTCGTTCCTCAACGTCGCCGACGCGGAGCGCATC      180
II              ATCACGGGGGAATTCGAGATGATGCAGTCGTTCCTCAACGTCGCCGACGCGGAGCGCATC      180
III             ATCACGGGGGAATTCGAGATGATGCAGTCGTTCCTCAACGTCGCCGACGCGGAGCGCATC      180
                ************************************************************

IV              AAGAACAACGCCGTCAAGACGTGGGTGCGGCAGCTCCGCGACCTGGCCTACGACACCGAA      240
V               AAGAACAACGCCGTCAAGACGTGGGTGCGGCAGCTCCGCGACCTGGCCTACGACACCGAA      240
I(Rwt4NLR)      AAGAACAACGCCGTCAAGACGTGGGTGCGGCAGCTCCGCGACCTGGCCTACGACACCGAA      240
II              AAGAACAACGCCGTCAAGACGTGGGTGCGGCAGCTCCGCGACCTGGCCTACGACACCGAA      240
III             AAGAACAACGCCGTCAAGACGTGGGTGCGGCAGCTCCGCGACCTGGCCTACGACACCGAA      240
                ************************************************************

IV              GACTGCATCGAGCTGGTGGTCCACCTGGATCCCAAGCCCAGATGGTGGCGCCGCCTGCTG      300
V               GACTGCATCGAGCTGGTGGTCCACCTGGATCCCAAGCCCAGATGGTGGCGCCGCCTGCTG      300
I(Rwt4NLR)      GACTGCATCGAGCTGGTGGTCCACCTGGATCCCAAGCCCAGATGGTGGCGCCGCCTGCTG      300
II              GACTGCATCGAGCTGGTGGTCCACCTGGATCCCAAGCCCAGATGGTGGCGCCGCCTGCTG      300
III             GACTGCATCGAGCTGGTGGTCCACCTGGATCCCAAGCCCAGATGGTGGCGCCGCCTGCTG      300
                ************************************************************

IV              GTCCTGCCTTGCCTGCCGGCGGTGTCGCTGCCCATGGACGACGCGGCGGCGGAGATAAAG      360
V               GTCCTGCCTTGCCTGCCGGCGGTGTCGCTGCCCATGGACGACGCGGCGGCGGAGATAAAG      360
I(Rwt4NLR)      GTCCTGCCTTGCCTGCCGGCGGTGTCGCTGCCCATGGACAACGCGGCGGCGGAGATAAAG      360
II              GTCCTGCCTTGCCTGCCGGCGGTGTCGCTGCCCATGGACGACGCGGCGGCGGAGATAAAG      360
III             GTCCTGCCTTGCCTGCCGGCGGTGTCGCTGCCCATGGACGACGCGGCGGCGGAGATAAAG      360
                *************************************** ********************

IV              GAGCTCAAGGACCGGGTGGAGTTCGTGAGCCAGAGGAACATGCGCTACAAGCTCATCACC      420
V               GAGCTCAAGGACCGGGTGGAGTTCGTGAGCCAGAGGAACATGCGCTACAAGCTCATCACC      420
I(Rwt4NLR)      GAGCTCAAGGACCGGGTGGAGTTCGTGAGCCAGAGGAACATGCGCTACAAGCTCATCACC      420
II              GAGCTCAAGGACCGGGTGGAGTTCGTGAGCCAGAGGAACATGCGCTACAAGCTCATCACC      420
III             GAGCTCAAGGACCGGGTGGAGTTCGTGAGCCAGAGGAACATGCGCTACAAGCTCATCACC      420
                ************************************************************

IV              GACTTCGGCGGCGCCAAGTCCGTCGCGCAGCAGCAGCTTGACCGGGCCGCCGGAGCTCTC      480
V               GACTTCGGCGGCGCCAAGTCCGTCGCGCAGCAGCAGCTTGACCGGGCCGCCGGAGCTCTC      480
I(Rwt4NLR)      GACTTCGGCGGCGCCAAGTCCGTCGCGCAGCAGCAGCTTGACCGGGCCGCCGGAGCTCTC      480
II              GACTTCGGCGGCGCCAAGTCCGTCGCGCAGCAGCAGCTTGACCGGGCCGCCGGAGCTCTC      480
III             GACTTCGGCGGCGCCAAGTCCGTCGCGCAGCAGCAGCTTGACCGGGCCGCCGGAGCTCTC      480
                ************************************************************

IV              GACATCGCCGTGGAGGAGGCGGCGAAGAAAGGTCGTAGCTTGGTGGATCTGACCGAGTTG      540
V               GACATCGCCGTGGAGGAGGCGGCGAAGAAAGGTCGTAGCTTGGTGGATCTGACCGAGTTG      540
I(Rwt4NLR)      GACATCGCCGTGGAGGAGGCGGCGAAGAAAGGTCGTAGCTTGGTGGATCTGACCGAGTTG      540
II              GACATCGCCGTGGAGGAGGCGGCGAAGAAAGGTCGTAGCTTGGTGGATCTGACCGAGTTG      540
III             GACATCGCCGTGGAGGAGGCGGCGAAGAAAGGTCGTAGCTTGGTGGATCTGACCGAGTTG      540
                ************************************************************

IV              ATCCCCAAAATGGAAGACCGCCCTGAGCTCGGCGTGATCTCGGTGTGGGGGACCGGCGGC      600
V               ATCCCCAGAATGGAAGACCGCCCTGAGCTCGGCGTGATCTCGGTGTGGGGGACCGGCGGC      600
I(Rwt4NLR)      ATCCCCAGAATGGAAGACCGCCCTGAGCTCGGCGTGATCTCGGTGTGGGGGACCGGCGGC      600
II              ATCCCCAGAATGGAAGACCGCCCTGAGCTCGGCGTGATCTCGGTGTGGGGGACCGGCGGC      600
III             ATCCCCAAAATGGAAGACCGCCCTGAGCTCGGCGTGATCTCGGTGTGGGGGACCGGCGGC      600
                ******* ****************************************************

IV              GACCTTGGGGTGGCGTCCATCGTCAGGAAGGCCTACGACGACCCTGAAATTTGCAAGAAC      660
V               GACCTTGGGGTGGCGTCCATCGTCAGGAAGGCCTACGACGACCCTGAAATTTGCAAGAAC      660
```

```
I(Rwt4NLR)      GACCTTGGGGTGGCGTCCATCGTCAGGAAGGCCTACGACGACCCTGAAATTTGCAAGAAC      660
II              GACCTTGGGGTGGCGTCCATCGTCAGGAAGGCCTACGACGACCCTGAAATTTGCAAGAAC      660
III             GACCTTGGGGTGGCGTCCATCGTCAGGAAGGCCTACGACGACCCTGAAATTTGCAAGAAC      660
                ************************************************************

IV              TTCCATAGTCGTGGCTGGGCCAAGTTGACCCACCCTTTCGATCCGCGGAAGATCCTCCGG      720
V               TTCCATAGTCGTGGCTGGGCCAAGTTGACCCACCCTTTCGATCCGCGGAAGATCCTCCGG      720
I(Rwt4NLR)      TTCCATAGTCGTGGCTGGGCCAAGTTGACCCACCCTTTCGATCCGCGGAAGATCCTCCGG      720
II              TTCCATAGTCGTGGCTGGGCCAAGTTGACCCACCCTTTCGATCCGCGGAAGATCCTCCGG      720
III             TTCCATAGTCGTGGCTGGGCCAAGTTGACCCACCCTTTCGATCCGCGGAAGATCCTCCGG      720
                ************************************************************

IV              AGCTTGTTCATTCAGTTCTGCACTAACACTGCTGCTCCACAGAAGCAAGGGGAAACTCTG      780
V               AGCTTGTTCATTCAGTTCTGCACTAACACTGCTGCTCCACAGAAGCAAGGGGAAACTCTG      780
I(Rwt4NLR)      AGCTTGTTCATTCAGTTCTGCACTAACACTGCTGCTCCACAGAAGCAAGGGGAAACTCTG      780
II              AGCTTGTTCATTCAGTTCTGCACTAACACTGCTGCTCCACAGAAGCAAGGGGAAACTCTG      780
III             AGCTTGTTCATTCAGTTCTGCACTAACACTGCTGCTCCACAGAAGCAAGGGGAAACTCTG      780
                ************************************************************

IV              GATGTAGATTCCCTTCTAAGGATGGAGAAGACGGTGGTGGAAGAAGGGGAGCTGGTCAAG      840
V               GATGTAGATTCCCTTCTAAGGATGGAGAAGACGGTGGTGGAAGAAGGGGAGCTGGTCAAG      840
I(Rwt4NLR)      GATGTAGATTCCCTTCTAAGGATGGAGAAGACGGTGGTGGAAGAAGGGGAGCTGGTCAAG      840
II              GATGTAGATTCCCTTCTAAGGATGGAGAAGACGGTGGTGGAAGAAGGGGAGCTGGTCAAG      840
III             GATGTAGATTCCCTTCTAAGGATGGAGAAGACGGTGGTGGAAGAAGGGGAGCTGGTCAAG      840
                ************************************************************

IV              CAGTTTGTGAGTCATGTGAGCACCCACAGGTTCCTCGTTGTCCTCGAAGGCGTGTCAACC      900
V               CAGTTTGTGAGTCATGTGAGCACCCACAGGTTCCTCGTTGTCCTCGAAGGCGTGTCAACC      900
I(Rwt4NLR)      CAGTTTGTGAGTCATGTGAGCACCCACAGGTTCCTCGTTGTCCTCGAAGGCGTGTCAACC      900
II              CAGTTTGTGAGTCATGTGAGCACCCACAGGTTCCTCGTTGTCCTCGAAGGCGTGTCAACC      900
III             CAGTTTGTGAGTCATGTGAGCACCCACAGGTTCCTCGTTGTCCTCGAAGGCGTGTCAACC      900
                ************************************************************

IV              ATGGCGGAATGGGATGCCCTCCGGATGTACCTGCCAGACATGGGTAACGGGAGCCAGATC      960
V               ATGGCGGAATGGGATGCCCTCCGGATGTACCTGCCAGACATGGGTAACGGGAGCCAGATC      960
I(Rwt4NLR)      ATGGCGGAATGGGATGCCCTCCGGATGTACCTGCCAGACATGGGTAACGGGAGCCAGATC      960
II              ATGGCGGAATGGGATGCCCTCCGGATGTACCTGCCAGACATGGGTAACGGGAGCCAGATC      960
III             ATGGCGGAATGGGATGCCCTCCGGATGTACCTGCCAGACATGGGTAACGGGAGCCAGATC      960
                ************************************************************

IV              ATCGTATCCACACAGCATTTTGACATCGCGAGCTTGTGCACCGGGCAGCTGCACAAAGTT      1020
V               ATCGTATCCACACAGCATTTTGACATCGCGAGCTTGTGCACCGGGCAGCTGCACAAAGTT      1020
I(Rwt4NLR)      ATCGTATCCACACAGCATTTTGACATCGCGAGCTTGTGCACCGGGCAGCTGCACAAAGTT      1020
II              ATCGTATCCACACAGCATTTTGACATCGCGAGCTTGTGCACCGGGCAGCTGCACAAAGTT      1020
III             ATCGTATCCACACAGCATTTTGACATCGCGAGCTTGTGCACCGGGCAGCTGCACAAAGTT      1020
                ************************************************************

IV              TCAGAGTTGAGAAAATTCACACCTGACCACTCTGTTTGTGTCTTTTTCAAGGAAGTATGT      1080
V               TCAGAGTTGAGAAAATTCACACCTGACCACTCTGTTTGTGTCTTTTTCAAGGAAGTATGT      1080
I(Rwt4NLR)      TCAGAGTTGAGAAAATTCACACCTGACCACTCTGTTTGTGTCTTTTTCAAGGAAGTATGT      1080
II              TCAGAGTTGAGAAAATTCACACCTGACCACTCTGTTTGTGTCTTTTTCAAGGAAGTATGT      1080
III             TCAGAGTTGAGAAAATTCACACCTGGCCACTCTGTTTGTGTCTTTTTCAAGGAAGTATGT      1080
                ************************* **********************************

IV              AATTAAGCACACCGTGACACTTTATTCACTTGTCATTTATTCATTGTCTGAAGAAATGCA      1140
V               AATTAAGCACACCGTGACACTTTATTCACTTGTCATTTATTCATTGTCTGAAGAAATGCA      1140
I(Rwt4NLR)      AATTAAGCACACCGTGACACTTTATTCACTTGTCATTTATTCATTGTCTGAAGAAATGCA      1140
II              AATTAAGCACACCGTGACACTTTATTCACTTGTCATTTATTCATTGTCTGAAGAAATGCA      1140
III             AATTAAGCACACCGTGACACTTTATTCACTTGTCATTTATTCATTGTCTGAAGAAATGCA      1140
                ************************************************************

IV              ACGCTGGAGGTGTAACTTTTGTTGATTTGTCAGGTTTCCGGAGGTGATGATCCTGGAAAT      1200
V               ACGCTGGAGGTGTAACTTTTGTTGATTTGTCAGGTTTCCGGAGGTGATGATCCTGGAAAT      1200
I(Rwt4NLR)      ACGCTGGAGGTGTAACTTTTGTTGATTTGTCAGGTTTCCGGAGGTGATGATCCTGGAAAT      1200
II              ACGCTGGAGGTGTAACTTTTGTTGATTTGTCAGGTTTCCGGAGGTGATGATCCTGGAAAT      1200
III             ACGCTGGAGGTGTAACTTTTGTTGATTTGTCAGGTTTCCGGAGGTGATGATCCTGGAAAT      1200
                ************************************************************

IV              GCAATGTTGGAGGGAGATGACGAAACTGAACTTATTGGGCGCGACACAGAAGAGGACGAG      1260
V               GCAATGTTGGAGGGAGATGACGAAACTGAACTTATTGGGCGCGACACAGAAGAGGACGAG      1260
I(Rwt4NLR)      GCAATGTTGGAGGGAGATGACGAAACTGAACTTATTGGGCGCGACACAGAAGAGGACGAG      1260
II              GCAATGTTGGAGGGAGATGACGAAACTGAACTTATTGGGCGCGACACAGAAGAGGACGAG      1260
III             GCAATGTTGGAGGGAGATGACGAAACTGAACTTATTGGGCGCGACACAGAAGAGGACGAG      1260
                ************************************************************

IV              CTTATGGAGCAGATTGATGAGGTTTCCCGTGTGATCTCCGTATGGGGGATTGCTGGTGTA      1320
V               CTTATGGAGCAGATTGATGAGGTTTCCCGTGTGATCTCCGTATGGGGGATTGCTGGTGTA      1320
```

```
I(Rwt4NLR)      CTTATGGAGCAGATTGATGAGGTTTCCCGTGTGATCTCCGTATGGGGGATTGCTGGTGTA      1320
II              CTTATGGAGCAGATTGATGAGGTTTCCCGTGTGATCTCCGTATGGGGGATTGCTGGTGTA      1320
III             CATATGGAGCAGATTGATGAGGTTTCCCGTGTGATCTCCGTATGGGGGATTGCTGGTGTA      1320
                * **********************************************************

IV              GGAAAATCGGCACTTGTCAGAAGTGCCTACTGCAAGTATCTGCAGGACCGCCCCAACTTC      1380
V               GGAAAATCGGCACTTGTCAGAAGTGCCTACTGCAAGTATCTGCAGGACCGCCCCAACTTC      1380
I(Rwt4NLR)      GGAAAATCGGCACTTGTCAGAAGTGCCTACTGCAAGTATCTGCAGGACCGCCCCAACTTC      1380
II              GGAAAATCGGCACTTGTCAGAAGTGCCTACTGCAAGTATCTGCAGGACCGCCCCAACTTC      1380
III             GGAAAATCGGCACTTGTCAGAAGTGCCTACTGCAAGTATCTGCAGGACCGCCCCAACTTC      1380
                ************************************************************

IV              GCCGGGTTAAATCTGACCTACATACAAGGGCGCGTGAACGTGCCCCATCCCTTCAATCTG      1440
V               GCCGGGTTAAATCTGACCTACATACACGGGCGCGTGAACGTGCCCCATCCCTTCAATCTG      1440
I(Rwt4NLR)      GCCGGGTTAAATCTGACCTACATACACGGGCGCGTGAACGTGCCCCATCCCTTCAATCTG      1440
II              GCCGGGTTAAATCTGACCTACATACACGGGCGCGTGAACGTGCCCCATCCCTTCAATCTG      1440
III             GCCGGGTTAAATCTGACCTACATACACGGGCGCGTGAACGTGCCCCATCCCTTCAATCTG      1440
                ************************** *********************************

IV              AGGGAGTTCTGCCGCAGCTTGCTCCTGAACTTGTCCTCTGAGCCTGCTAAAACCAAAGAG      1500
V               AGGGAGTTCTGCCGCAGCTTGCTCCTGAACTTGTCCTCTGAGCCTGCTAAAACCAAAGAG      1500
I(Rwt4NLR)      AGGGAGTTCTGCCGCAGCTTGCTCCTGAACTTGTCCTCTGAACCTGCTAAAACCAAAGAG      1500
II              AGGGAGTTCTGCCGCAGCTTGCTCCTGAACTTGTCCTCTGAGCCTGCTAAAACCAAAGAG      1500
III             AGGGAGTTCTGCCGCAGCTTGCTCCTGAACTTGTCCTCTGAGCCTGCTAAAACCAAAGAG      1500
                ***************************************** ******************

IV              GACGCCGTCGCTGAACTGGCAAACATGACCAATCCAATTGATGTCTGCCGTAAGCTTCTT      1560
V               GACGCCGTCGCTGAACTGGCAAACATGACCAATCCAATTGATGTCTGCCGTAAGCTTCTT      1560
I(Rwt4NLR)      GACGCCGTCGCTGAACTGGCAAACATGACCAATCCAATTGATGTCTGCCGTAAGCTTCTT      1560
II              GACGCCGTCGCTGAACTGGCAAACATGACCAATCCAATTGATGTCTGCCGGAAGCTTCTT      1560
III             GACGCCGTCGCTGAACTGGCAAACATGACCAATCCAATTGATGTCTGCCGTAAGCTTCTT      1560
                ************************************************** *********

IV              AGCGACGATCAACAACAGAAGCAGTGCCTTATTGTTATTGATGACCTGCAGTCCACTGAA      1620
V               AGCGACGATCAACAACAGAAGCAGTGCCTTATTGTTATTGATGACCTGCAGTCCACTGAA      1620
I(Rwt4NLR)      AGCGACGATCAACAGCAGAAGCAGTGCCTTATTGTTATTGATGACCTGCAGTCCACTGAA      1620
II              AGCGACGATCAACAACAGAAGCAGTGCCTTATTGTTATTGATGACCTGCAGTCCACTGAA      1620
III             AGCGACGATCAACAACAGAAGCAGTGCCTTATTGTTATTGATGACCTGCAGTCCACTGAA      1620
                ************** *********************************************

IV              GAATGGGACAGAATAAAACAAGCCTTGTCATTTGGTAAAGAATGTTGTATCATAGTTGTT      1680
V               GAATGGGACAGAATAAAACAAGCCTTGTCATTTGGTAAAGAATGTTGTATCATAGTTGTT      1680
I(Rwt4NLR)      GAATGGGACAGAATAAAACAAGCCTTGTCATTTGGTAAAGAATGTTGTATCATAGTTGTT      1680
II              GAATGGGACAGAATAAAACAAGCCTTGTCATTTGGTAAAGAATGTTGTATCATAGTTGTT      1680
III             GAATGGGACAGAATAAAACAAGCCTTGTCATTTGGTAAAGAATGTTGTATCATAGTTGTT      1680
                ************************************************************

IV              ACAAATGATTCAAGTGTTGCCAAATATTGTTCAGGGCAAGATCAGAAACTCATGTTCAAC      1740
V               ACAAATGATTCAAGTGTTGCCAAATATTGTTCAGGGCAAGATCAGAAACTCATGTTCAAC      1740
I(Rwt4NLR)      ACAAATGATTCAAGTGTTGCCAAATATTGTTCAGGGCAAGATCAGAAACTCATGTTCAAC      1740
II              ACAAATGATTCAAGTGTTGCCAAATATTGTTCAGGGCAAGATCAGAAACTCATGTCCAAC      1740
III             ACAAATGATTCAAGTGTTGCCAAATATTGTTCAGGGCAAGATCAGAAACTCATGTTCAAC      1740
                ******************************************************* ****

IV              GTCAAAGGTCTTGGTGACGAGCATGCCCCTTGACCTCTTCAACACGGTACTCGCTGCTCTA      1800
V               GTCAAAGGTCTTGGTGACGAGCATGCCCCTTGACCTCTTCAACACGGTACTCGCTGCTCTA      1800
I(Rwt4NLR)      GTCAAAGGTCTTGGTGACGAGCATGCCCCTTGACCTCTTCAACACGGTACTCGCTGCTCTA      1800
II              GTCAAAGGTCTTGGTGACGAGCATGCCCCTTGACCTCTTCAACATGGTACTCGCTGCTCTA      1800
III             GTCAAAGGTCTTGGTGACGAGCATGCCCCTTGACCTCTTCAACACGGTACTCGCTGCTCTA      1800
                ******************************************** ***************

IV              AAATAACCATTCCTGCAAATTCAACTCCTTATTAGAGTCCTATATTTACTTTTTCTGTAT      1860
V               AAATAACCATTCCTGCAAATTCAACTCCTTATTAGAGTCCTATATTTACTTTTTCTGTAT      1860
I(Rwt4NLR)      AAATAACCATTCCTGCAAATTCAACTCCTTATTAGAGTCCTATATTTACTTTTTCTGTAT      1860
II              AAATAACCATTCCTGCAAATTCAACTCCTTATTAGAGTCCTATATTTACTTTTTCTGTAT      1860
III             AAATAACCATTCCTGCAAATTCAACTCCTTATTAGAGTCCTATATTTACTTTTTCTGTAT      1860
                ************************************************************

IV              ACCACAGCACGGATCTTCAGTACACCCAGATGTAGCTATAGAGCATGTTTGGTTCATAGT      1920
V               ACCACAGCACGGATCTTCAGTACACCCAGATGTAGCTATAGAGCATGTTTGGTTCATAGT      1920
I(Rwt4NLR)      ACCACAGCACGGATCTTCAGT---CCCAGATGTAGCTATAGAGCATGTTTGGTTCATAGT      1917
II              ACCACAGCACGGATCTTCAGTACACCCAGATGTAGCTATAGAGCATGTTTGGTTCATAGT      1920
III             ACCACAGCACGGATCTTCAGTACACCCAGATGTAGCTATAGAGCATGTTTGGTTCATAGT      1920
                *********************   ************************************

IV              CATACTCATACCGTGTCTAATTTTGCCACTTTCTTGTATAGCAGCCTGTATCCTCAGCCA      1980
V               CATACTCATACCGTGTCTAATTTTGCCACTTTCTTGTATAGCAGCCTGTATCCTCAGCCA      1980
```

```
I(Rwt4NLR)     CATACTCATACCGTGTCTAATTTTGCCACTTTCTTGTATAGCAGCCTGTATCCTCAGCCA     1977
II             CATACTCATACCGTGTCTAATTTTGCCACTTTCTTGTATAGCAGCCTGTATCCTCAGCCA     1980
III            CATACTCATACCGTGTCTAATTTTGCCACTTTCTTGTATAGCAGCCTGTATCCTCAGCCA     1980
               ************************************************************

IV             GTCTCATGCTTTCGCAAAACCGGGGGCAAAGTATTGACTGCCAACTTCTTTGTCGGACTG     2040
V              GTCTCATACTTTCGCAAAACCGGGGGCAAAGTATTAACTGCCAACTTCTTTGTCGGACTG     2040
I(Rwt4NLR)     GTCTCACACTTTCGCAAAACCGGGGGCAAAGTATTAACTGCCAACTTCTTTGTCGGACTG     2037
II             GTCTCATACTTTCGCAAAACCGGGGGCAAAGTATTAACTGCCAACTTCTTTGTCGGACTG     2040
III            GTCTCATACTTTCGCAAAACCGGGGGCAAAGTATTAACTGCCAACTTCTTTGTCGGACTG     2040
               ******  *************************** ************************

IV             AATAAAAATGTATCAAACTTAAGTCATGCTTTTTGGAACACGGCAAAAACAACATGGTAT     2100
V              AATAAAAATGTATCAAACTTAAGTCATGCTTTTTGGAACACGGCAAAAACAACATGGTAT     2100
I(Rwt4NLR)     AATAAAAATGTATCAAACTTAAGTCATGCTTTTTGGAACACGGCAAAAACAACATGGTAT     2097
II             AGTAAAAATGTATCAAACTTAAGTCATGCTTTTTGGAACACGGCAAAAACAACATGGTAT     2100
III            AATAAAAATGTATCAAACTTAAGTCATGCTTTTTGGAACACGGCAAAAACAACATGGTAT     2100
               * **********************************************************

IV             ACATACAACCAGTACAGGAACTTATGTGATGGTTAGCAGGATAGTATAGGAATAATGAAG     2160
V              ACATACAACCAGTACAGGAACTTATGTGATGGTTAGCAGGATAGTATAGGAATAATGAAG     2160
I(Rwt4NLR)     ACATACAACCAGTACAGGAACTTATGTGATGGTTAGCAGGATAGTATAGGAATAATGAAG     2157
II             ACATACAACCAGTACAGGAACTTATGTGATGGTTAGCAGGATAGTATAGGAATAATGAAG     2160
III            ACATACAACCAGTACAGGAACTTATGTGATGGTTAGCAGGATAGTATAGGAATAATGAAG     2160
               ************************************************************

IV             GCATGAATTAGCCAGAAGCGGTGTTTGAATGCTTCAGGGGAAAAATAGTTACATATTTGT     2220
V              GCATGAATTAGCCAGAAGCGGTGTTTGAATGCTTCAGGGGAAAAATAGTTACATATTTGT     2220
I(Rwt4NLR)     GCATGAATTAGCCAGAAGCGGTGTTTGAATGCTTCAGGGGAAAAATAGTTACATATTTGT     2217
II             GCATGAATTAGCCAGAAGCGGTGTTTGAATGCTTCAGGGGAAAAATAGTTACATATTTGT     2220
III            GCATGAATTAGCCAGAAGCGGTGTTTGAATGCTTCAGGGGAAAAATAGTTACATATTTGT     2220
               ************************************************************

IV             AAAGTACCTTCCAAAATTTATAGAAAGTAATTCTTCTATGCAGTTGTACATTTCAAAGAC     2280
V              AAAGTACCTTCCAAAATTTATAGAAAATAATTCTTCTATGCAGTTGTACATTTCAAAGAC     2280
I(Rwt4NLR)     AAAGTACCTTCCAAAATTTATAGAAAATAATTCTTCTATGCAGTTGTACATTTCAAAGAC     2277
II             AAAGTACCTTCCAAAATTTATAGAAAATAATTCTTCTATGCAGTTGTACATTTCAAAGAC     2280
III            AAAGTACCTTCCAAAATTTATAGAAAATAATTCTTCTATGCAGTTGTACATTTCAAAGAC     2280
               ************************** *********************************

IV             CGTGACTAACTATGTCAAGTTTGGCCACTTTTCAATAAAATTCCGGTAGCTAGGGAACCC     2340
V              CGTGACTAACTATGTCAAGTTTGGCCACTTTTCAATAAAATTCCGGTAGCTAGGGAACCC     2340
I(Rwt4NLR)     CGTGACTAACTATGTCAAGTTTGGCCACTTTTCAATAAAATTCCGGTAGCTAGGGAACCC     2337
II             CGTGACTAACTATGTCAAGTTTGGCCACTTTTCAACAAAATTCCGGTAGCTAGGGAACCC     2340
III            CGTGACTAACTATGTCAAGTTTGGCCACTTTTCAATAAAATTCCGGTAGCTAGGGAACCC     2340
               *********************************** ************************

IV             TCTCTATCCCCCATGACCATTCGGAAAGAAAAACGACCCTAGCTATTTGGAACTCTCCTT     2400
V              TCTCTATCCCCCATGACCATTCGGAAAGAAAAACGACCCTAGCTATTTGGAACTCTCCTT     2400
I(Rwt4NLR)     TCTCTATCCCCCATGACCATTCGGAAAGAAAAACGACCCTAGCTATTTGGAACTCTCCTT     2397
II             TCTCTATCCCCCATGACCATTCGGAAAGAAAAACGACCCTAGCTATTTGGAACTCTCCTT     2400
III            TCTCTATCCCCCATGACCATTCGGAAAGAAAAACGACCCTAGCTATTTGGAACTCTCCTT     2400
               ************************************************************

IV             ATGACTGAATTCTCAAATTATAAACAATGCTCATTGCTCGTAATTAACATGTCAAGGTCA     2460
V              ATGACTGAATTCTCAAATTATAAACAATGCTCATTGCTCGTAATTAACATATCAAGGTCA     2460
I(Rwt4NLR)     ATGACTGAATTCTCAAATTATAAACAATGCTCATTGCTCGTAATTAACATATCAAGGTCA     2457
II             ATGACTGAATTCTCAAATTATAAACAATGCTCATTGCTCGTAATTAACATATCAAGGTCA     2460
III            ATGACTGAATTCTCAAATTATAAACAATGCTCATTGCTCGTAATTAACATGTCAAGGTCA     2460
               ************************************************** *********

IV             GATGCCAAGAAATAAGATTAGGATACCAGCGTTACTCTCTCTGTTCTAAAATAGATTATT     2520
V              GATGCCAAGAAATAAGATTAGGATACCAGCGTTACTCTCTCTGTTTTAAAATAGATTATT     2520
I(Rwt4NLR)     GATGCCAAGAAATAAGATTAGGATACCAGCGTTACTCTCTCTGTTCTAAAATAGATTATT     2517
II             GATGCCAAGAAATAAGATTAGGATACCAGCGTTACTCTCTCTGTTCTAAAATAGATTATT     2520
III            GATGCCAAGAAATAAGATTAGGATACCAGCGTTACTCTCTCTGTTCTAAAATAGATTATT     2520
               ********************************************* **************

IV             CATTCTAAGATGACTTTATCAACGATTCAATGTTGCACGATTTTCACTACACAACTTTGA     2580
V              CATTCTAAGATGACTTTATCAACGATTCAATGTTGCACGATTTTCACTACACAACTTTGA     2580
I(Rwt4NLR)     CATTCTAAGATGACTTTATCAACGATTCAATGTTGCACGATTTTCACTACACAACTTTGA     2577
II             CATTCTAAGATGACTTTATCAACGATTCAATGTTGCACGATTTTCACTACACAACTTTGA     2580
III            CATTCTAAGATGACTTTATCAACGATTCAATGTTGCACGATTTTCACTACACAACTTTGA     2580
               ************************************************************

IV             TTGTTATCATTTGGCAAGGCTATGTAACAGAAAAATAGTTTTCATTAAAAAACTAATGAT     2640
V              TTGTTATCATTTGGCAAGGCTATGTAACAGAAAAATAGTTTTCATTAAAAAACTAATGAT     2640
```

```
I(Rwt4NLR)      TTGTTATCATTTGGCAAGGCTATGTAACAGAAAAATAGTTTTCATTAAAAAACTAATGAT      2637
II              TTGTTATCATTTGGCAAGGCTATGTAACAGAAAAATAGTTTTCATTAAAAAACTAATGAT      2640
III             TTGTTATCATTTGGCAAGGCTATGTAACAGAAAAATAGTTTTCATTAAAAAACTAATGAT      2640
                ************************************************************

IV              ATCATTCTGTGTGATGAGAAGTGTTTT-TTTATTAATCAGTGGTCATAGATAGAGAAGTT      2699
V               ATCATTCTGTGTGATGAGAAGTGTTTTTTTTATTAATCAGTGGTCATAGATAGAGAAGTT      2700
I(Rwt4NLR)      ATCATTCTGTGTGATGAGAAGTGTTTT-TTTATTAATCAGTGGTCATAGATAGAGAAGTT      2696
II              ATCATTCTGTGTGATGAGAAGTGTTTT-TTTATTAATCAGTGGTCATAGATAGAGAAGTT      2699
III             ATCGTTCTGTGTGATGAGAAGTGTTTT-TTTATTAATCAGTGGTCATAGATAGAGAAGTT      2699
                *** *********************** ********************************

IV              TAGTTGGACAATTCCTAAAACCACAGTTTATTTTCAGGTATATGACGGCGTGGGTAACCA      2759
V               TAGTTGGACAATTCCTAAAACCACAGTTTATTTTCAGGTATATGACGGCATGGGTAACCA      2760
I(Rwt4NLR)      TAGTTGGACAATTCCTAAAACCACAGTTTATTTTCAGGTATATGACGGCGTGGGTAACCA      2756
II              TAGTTGGACAATTCCTAAAACCACAGTTTATTTTCAGGTATATGACGGCGTGGGTAACCA      2759
III             TAGTTGGACAATTCCTAAAACCACAGTTTATTTTCAGGTATATGACGGCGTGGGTAACCA      2759
                ************************************************* **********

IV              CATCACACGGGATGAGGACATGATCAATCAGTCGAAGCTAATGTTAAACAAATGCGGCGG      2819
V               CATCACACGGGATGAGGACATGATCAATCAGTCGAAGCTAATGTTAAACAAATGCGGCGG      2820
I(Rwt4NLR)      CATCACACGGGATGAGGACATGATCAATCAGTCGAAGCTAATGTTAAACAAATGCGGCGG      2816
II              CATCACACGGGATGAGGACATGATCAATCAGTCGAAGCTAATGTTAAACAAATGCGGCGG      2819
III             CATCACACGGGATGAGGTCATGATCAATCAGTCGAAGCTAATGTTAAACAAATGCGGCGG      2819
                ***************** ******************************************

IV              ACTACCCAAGGTGATAGTTGCAATTGGTCGTTTCTTGGCCAAGAGTCTGAATTGGGAGGC      2879
V               ACTACCCAAGGTGATAGTTGCAATTGGTCGTTTCTTGGCCAAGAGTCTGAATTGGGAGGC      2880
I(Rwt4NLR)      ACTACCCAAGGTGATAGTTGCAATTGGTTGTTTCTTGGCCAAGAGTCTGAATTGGGAGGC      2876
II              ACTACCCAAGGTGATAGTTGCAATTGGTCGTTTCTTGGCCAAGAGTCTGAATTGGGAGGC      2879
III             ACTACCCAAGGTGATAGTTGCAATTGGTCGTTTCTTGGCCAAGAGTCTGAATTGGGAGGC      2879
                **************************** *******************************

IV              CATGAATACTAACTTTATTCACCAGTTGGAAAACAACCCGGAGCTGGCTAGTGTACATAG      2939
V               CATGAATACTAACTTTATTCACCAGTTGGAAAACAACCCGGAGCTGGCTAGTGTACATAG      2940
I(Rwt4NLR)      CATGAATACTAACTTTATTCACCAGTTGGAAAACAACCCGGAGCTGGCTAGTGTACATAG      2936
II              CATGAATACTAACTTTATTCACCAGTTGGAAAACAACCCGGAGCTGGCTAGTGTACATAG      2939
III             CATGAATACTAACTTTATTCACCAGTTGGAAAACAACCCGGAGCTGGCTAGTGTACATAG      2939
                ************************************************************

IV              CATGTTTAGTTGGCTAGACTCCTACTTCCACAATTGTCCTGACGAACTCAAGCCATGCAT      2999
V               CATGTTTAGTTGGCTAGACTCCTACTTCCACAATTGTCCTGACGAACTCAAGCCATGCAT      3000
I(Rwt4NLR)      CATGTTTAGTTGGCTAGACTCCTACTTCCACAATTGTCCTGACGAACTCAAGCCATGCAT      2996
II              CATGTTTAGTTGGCTAGACTCCTACTTCCACAATTGTCCTGACGAACTCAAGCCATGCAT      2999
III             CATGTTTAGTTGGCTAGACTCCTACTTCCACAATTGTCCTGACGAACTCAAGCCATGCAT      2999
                ************************************************************

IV              CTTCTATTTGTCAATCTTCCCTCGACGCCATGGCATTCGTCGAAGGCGTCTGGTGAGGCG      3059
V               CTTCTATTTGTCAATCTTCCCTCGACGCCATGGCATTCGTCGAAGGCGTCTGGTGAGGCG      3060
I(Rwt4NLR)      CTTCTATTTGTCAATCTTCCCTCGACGCCATGGCATTCGTCGAAGGCGTCTGGTGAGGCG      3056
II              CTTCTATTTGTCAATCTTCCCTCGACGCCATGGCATTCGTCGAAGGCGTCTGGTGAGGCG      3059
III             CTTCTATTTGTCAATCTTCCCTCGACGCCATGGCATTCGTCGAAGGCGTCTGGTGAGGCG      3059
                ************************************************************

IV              GTGGATTGCCGAGGGCTACTCAAGAGACACCGACGGGAATCTCGCGGAAGTGAACGGGGA      3119
V               GTGGATTGCCGAGGGCTACTCAAGAGACACCGACGGGAATCTCGCGGAAGTGAACGGGGA      3120
I(Rwt4NLR)      GTGGATTGCCGAGGGCTACTCAAGAGACACCGACGGGAATCTCGCGGAAGTGAACGGGGA      3116
II              GTGGATTGCCGAGGGCTACTCAAGAGACACCGACGGGAATCTCGCGGAAGTGAACGGGGA      3119
III             GTGGATTGCCGAGGGCTACTCAAGAGACACCGACGGGAATCTCGCGGAAGTGAACGGGGA      3119
                ************************************************************

IV              AAATTACTTCTCCAGGCTCGTCAACCTCAGCATGCTCACAGATGCTGAGCGTGATGTCAA      3179
V               AAATTACTTCTCCAGGCTCGTCAACCTCAGCATGCTCACAGATGCTGAGCGTGATGTCAA      3180
I(Rwt4NLR)      AAATTACTTCTCCAGGCTCGTCAACCTCAGCATGCTCACAGATGCTGAGCGTGATGTCAA      3176
II              AAATTACTTCTCCAGGCTCGTCAACCTCAGCATGCTCACAGATGCTGAGCGTGATGTCAA      3179
III             AAATTACTTCTCCAGGCTCGTCAACCTCAGCATGCTCACAGATGCTGAGCGTGATGTCAA      3179
                ************************************************************

IV              CGTGGCTGGGACAGCGACTGGGGCTGGTACTGGGAGGAGAATGGCCATGTGCAACGTCAA      3239
V               CGTGGCTGGGACAGCGACTGGGGCTGGTACTGGGAGGAGAATGGCCATGTGCAACGTCAA      3240
I(Rwt4NLR)      CGTGGCTGGGACAGCGACTGGGGCTGGTACTGGGAGGAGAATGGCCATGTGCAACGTCAA      3236
II              CGTGGCTGGGACAGCGACTGGGGCTGGTACTGGGAGGAGAATGGCCATGTGCAACGTCAA      3239
III             CGTGGCTGGGACAGCGACTGGGGCTGGTACTGGGAGGAGAATGGCCATGTGCAACATCAA      3239
                ******************************************************* ****

IV              CGATTTCTTCCGGGAGTACATTGTGTCGCGGCGAATGGAGGAGAACCATGTGTTTGCACT      3299
V               CGATTTCTTCCGGGAGTACATTGTGTCGCGGCGAATGGAGGAGAACCATGTGTTTGCACT      3300
```

```
I(Rwt4NLR)      CGATTTCTTCCGGGAGTACATTGTGTCGCGGCGAATGGAGGAGAACCATGTGTTTGCACT      3296
II              CGATTTCTTCCGGGAGTACATTGTGTCGCGGCGAATGGAGGAGAACCATGTGTTTGCACT      3299
III             CGATTTCTTCCGGGAGTACATTGTGTCGCGGCGAATGGAGGAGAACCATGTGTTTGCACT      3299
                ************************************************************

IV              GGAGGGGAGGTGCAGCCAGACCACGCGGCGCACTGGACGGCACCTGGTCATCGACAAAAG      3359
V               GGAGGGGAGGTGCAGCCAGACCACGCGGCGCACTGGACGGCACCTGGTCATCGACAAAAG      3360
I(Rwt4NLR)      GGAGGGGAGGTGCAGCCAGACCACGCGGCGCACTGGACGGCACCTGGTCATCGACAAAAG      3356
II              GGAGGGGAGGTGCAGCCAGACCACGCGGCGCACTGGACGGCACCTGGTCATCGACAAAAG      3359
III             GGAGGGGAGGTGCAGCCAGACCACGCGGCGCACTGGACGGCACCTGGTCATCGACAAAAG      3359
                ************************************************************

IV              CTGGGACGGGGACGAGAGCGTGTTCAACAGGATCGAGTTCTCGCGACTGCGGTCGGTGAC      3419
V               CTGGGACGGGGACGAGAGCGTGTTCAACAGGATCGAGTTCTCGCGACTGCGGTCGGTGAC      3420
I(Rwt4NLR)      CTGGGACGGGGACGAGAGCGTGTTCAACAGGATCGAGTTCTCGCGACTGCGGTCGGTGAC      3416
II              CTGGGACGGGGACGAGAGCGTGTTCAACAGGATCGAGTTCTCGCGACTGCGGTCGGTGAC      3419
III             CTGGGACGGGGACGAGAGCGTGTTCAACAGGATCGAGTTCTCGCGACTGCGGTCGGTGAC      3419
                ************************************************************

IV              GGTTTTCGGACCCTGGAAGCCATTCCTGGCCTCCGACAAGATGAGGGTGCTCCGCGTGCT      3479
V               GGTTTTCGGACCCTGGAAGCCATTCCTGGCCTCCGACAAGATGAGGGTGCTCCGCGTGCT      3480
I(Rwt4NLR)      GGTGTTCGGACCCTGGAAGCCATTCCTGGCCTCCGACAAGATGAGGGTGCTCCGCGTGCT      3476
II              GGTGTTCGGACCCTGGAAGCCATTCCTGGCCTCCGACAAGATGAGGGTGCTCCGCGTGCT      3479
III             GGTGTTCGGACCCTGGAAGCCATTCCTGGCCTCCGACAAGATGAGGGTGCTCCGCGTGCT      3479
                *** ********************************************************

IV              TGATCTGGAAGGCACCGAGGGTCTGACAGACGACGACATCAAGAACATCGTCAAACGGCT      3539
V               TGATCTGGAAGGCACCGAGGGTCTGACAGACGACGACATCAAGAACATCGTCAAACGGCT      3540
I(Rwt4NLR)      TGATCTGGAAGGCACTGAGGGTCTGACAGACAACGACATCAAGAACATCGTCAAACGGCT      3536
II              TGATCTGGAAGGCACTGAGGGTCTGACAGACAACGACATCAAGAACATCGTCAAACGGCT      3539
III             TGATCTGGAAGGCACTGAGGGTCTGACAGACAACGACATCAAGAACATCGTCAAACGGCT      3539
                *************** *************** ****************************

IV              GCCTCGACTGAAGTTCCTCTCCCTGCGAGGATGCAAAAACATCTTTCGGCTGCCGAAATC      3599
V               GCCTCGACTGAAGTTCCTCTCCCTGCGAGGATGCAAAAACATCTTTCGGCTGCCGAAATC      3600
I(Rwt4NLR)      GCCTCGACTAAAGTTCCTCTCCCTGCGAGGATGCAAGCACATCTTTCGGCTGCCAAAATC      3596
II              GCCTCGACTAAAGTTCCTCTCCCTGCGAGGATGCAAGCACATCTTTCGGCTGCCAAAATC      3599
III             GCCTCGACTAAAGTTCCTCTCCCTGCGAGGATGCAAGCACATCTTTCGGCTGCCAAAATC      3599
                ********* **************************  **************** *****

IV              CCTTGGTCGCCTGAGGCAGCTCCAGACCCTGGATGTCAGGCACACTGCCATAGCCGGGCT      3659
V               CCTTGGTCGCCTGAGGCAGCTCCAGACCCTGGATGTCAGGCACACTGCCATAGCCGGGCT      3660
I(Rwt4NLR)      CCTGGGTCGCCTGAGGCAGCTCCAGACCCTGGATGTGAGGCACACTGCCGTAGCCGGGCT      3656
II              CCTGGGTCGCCTGAGGCAGCTCCAGACCCTGGATGTGAGGCACACTGCCGTAGCCGGGCT      3659
III             CCTGGGTCGCCTGAGGCAGCTCCAGACCCTGGATGTGAGGCACACTGCCGTAGCCGGGCT      3659
                *** ******************************** ************ **********

IV              GCCAGCGACCGTCGTGAAGCTACAGAAGCTGCAGTATGTTCGTGCCGGTAACACACCAGA      3719
V               GCCAGCGACCGTCGTGAAGCTACAGAAGCTGCAGTATGTTCGTGCCGGTAACACACCAGA      3720
I(Rwt4NLR)      GCCAGCGACCGTCGTGAAGCTACAGAAGCTGCAGTATGTTCGTGCCGGCAACACACCAGA      3716
II              GCCAGCGACCGTCGTGAAGCTACAGAAGCTGCAGTATGTTCGTGCCGGCAACACACCAGA      3719
III             GCCAGCGACCGTCGTGAAGCTACAGAAGCTGCAGTATGTTCGTGCCGGCAACACACCAGA      3719
                ************************************************ ***********

IV              GGAATATGCCGGCAGCGACAGCACGGAATCTAGCACCTCGGTACCCGCCTGTTTCTCATG      3779
V               GGAATATGCCGGCAGCGACAGCACGGAATCTAGCACCTCGGTACCCGCCTGTTTCTCATG      3780
I(Rwt4NLR)      GGAGTATGCCGTCAGCGACAGCACGGAATCTAGCACCTCAGTACCCACCTGTTTCTCATG      3776
II              GGAGTATGCCGTCAGCGACAGCACGGAATCTAGCACCTCAGTACCCACCTGTTTCTCATG      3779
III             GGAGTATGCCGTCAGCGACGGCACGGAATCTAGCACCTCGGTACCCACCTGTTTCTCATG      3779
                *** ******* ******* ******************* ****** *************

IV              CTGCCCTGTTGGCGGTCACATCGTCGGCGTTGCGGTGCCTCGCGGGGTCGAGAATCTGAC      3839
V               CTGCCCTGTTGGCGGTCACATCGTCGGCGTTGCGGTGCCTCGCGGGGTCGAGAATCTGAC      3840
I(Rwt4NLR)      TGGCCCTGTTGGCAATCACATTGTCGGCGTTGAGGTGCCTCGCGGGGTCGAGAATCTGAC      3836
II              TGGCCCTGTTGGCAATCACATTGTCGGCGTTGAGGTGCCTCGCGGGGTCGAGAATCTGAC      3839
III             TGGCCCTGTTGGCAATCACATTGTCGGCGTTGAGGTGCCTCGCGGGGTCGAGAATCTGAC      3839
                  ***********  ****** ********** ***************************

IV              CAACCTGCACACACTGGGCGTCATCAAAGCCACTGTGGCAGGACTCAAGGAGCTCAAGAA      3899
V               CAACCTGCACACACTGGGCGTCATCAAAGCCACTGTGGCAGGACTCAAGGAGCTCAAGAA      3900
I(Rwt4NLR)      CAACCTGCACACACTGGGCGTCATCAAAGCCACTGTGGCAGGACTCAAGGAGCTCAAGAA      3896
II              CAACCTGCACACACTGGGCGTCATCAAAGCCACTGTGGCAGGACTCAAGGAGCTCAAGAA      3899
III             CAACCTGCACACACTGGGCGTCATCAAAGCCACTGTGGCAGGACTCAAGGAGCTCAAGAA      3899
                ************************************************************

IV              GCTTACCCAGCTGCGCAAGCTCGGTGTGTCCGGCATAAACCGGAGTAACCACAAAGAGCT      3959
V               GCTTACCCAGCTGCGCAAGCTCGGTGTGTCCGGCATAAACCGGAGTAACCACAAAGAGCT      3960
```

```
I(Rwt4NLR)      GCTTACCCAGCTGCGCAAGCTCGGCGTGTCTGGCATAAACCGGAGGAACCACAAAGAGCT      3956
II              GCTTACCCAGCTGCGCAAGCTCGGCGTGTCTGGCATAAACCGGAGGAACCACAAAGAGCT      3959
III             GCTTACCCAGCTGCGCAAGCTCGGCGTGTCTGGCATAAACCGGAGGAACCACAAAGAGCT      3959
                ************************ ***** ************** **************

IV              CCGCGCCGTCGTCTCCGGACATGGTCATCTGGAATCCCTGTCGATATGGCTTGACAAGGA      4019
V               CCGCGCCGTCGTCTCCGGACATGGTCATCTGGAATCCCTGTCGATATGGCTTGACAAGGA      4020
I(Rwt4NLR)      CTGCGCCGTCATCTCGGGCCACGGCCATCTGGAATCTCTGTCGATATGGTTTGACAAGGA      4016
II              CTGCGCCGTCATCTCGGGCCACGGCCATCTGGAATCTCTGTCGATATGGTTTGACAAGGA      4019
III             CTGCGCCGTCGTCTCGGGCCACGGCCATCTGGAATCTCTGTCGATATGGTTTGACAAGGA      4019
                * ******** **** ** ** ** *********** ************ **********

IV              CACCGAGGGGATTGATGACCCTTGCTTGCCGATCAATGATGACTTCAAGGCTCCAGAGAA      4079
V               CACCGAGGGGATTGATGACCCTTGCTTGCCGATCAATGATGACTTCAAGGCTCCAGAGAA      4080
I(Rwt4NLR)      CACCGAGAGGATTGCTGACCCTTGTTCGCCGAGCAATGATGACTTCAAGCCTCCAGAGAA      4076
II              CACCGAGAGGATTGCTGACCCTTGTTCGCCGAGCAATGATGACTTCAAGCCTCCAGAGAA      4079
III             CACCGAGAGGATTGCTGACCCTTGTTCGCCGAGCAATGATGACTTCAAGCCTCCAGAGAA      4079
                ******* ****** ********* * ***** **************** **********

IV              GCTACGGAGGCTCAAACTCCACGGGCACATCGGCAACTTGCCAGCATGGGTTAGCACTCT      4139
V               GCTACGGAGGCTCAAACTCCACGGGCACATCGGCAACTTGCCAGCATGGGTTAGCACTCT      4140
I(Rwt4NLR)      GCTACGGAGGCTCAAACTCCATGGGCACACTGACAACCTGCCAGCATGGGTTAGCACTCT      4136
II              GCTACGGAGGCTCAAACTCCATGGGCACACTGACAACCTGCCAGCATGGGTTAGCACTCT      4139
III             GCTACGGAGGCTCAAACTCCATGGGCACACTGACAACCTGCCAGCATGGGTTAGCACTCT      4139
                ********************* *******  * **** **********************

IV              CA-------------------------- 4141
V               CA-------------------------- 4142
I(Rwt4NLR)      CAAAATATTTGTCACTCAAGATCAATGA 4164
II              CAAAATATTTGTCACTCAAGATCAATGA 4167
III             CAAAATATTTGTCACTCAAGATCAATGA 4167
                **
```
